# A rice class-XIV kinesin enters the nucleus in response to cold

**DOI:** 10.1038/s41598-018-21816-w

**Published:** 2018-02-26

**Authors:** Xiaolu Xu, Wilhelm J. Walter, Qiong Liu, Isabel Machens, Peter Nick

**Affiliations:** 1 Molecular Cell Biology, Botanical Institute, Karlsruhe Institute of Technology (KIT), Fritz-Haber-Weg 4, 76131 Karlsruhe, Germany; 2 Molecular Plant Physiology, Biocentre Klein Flottbek, University of Hamburg, 22609 Hamburg, Germany

## Abstract

Higher plants possess a large number of kinesins, but lack the minus-end directed dynein motors. However, the kinesin class XIV has strongly expanded, and minus-end directed motors from this class may have taken over functions of cytoplasmic dyneins. In this study, we address the functional aspects of a novel rice homologue of the *Arabidopsis* class-XIV kinesins ATK1 and ATK5. Since a loss-of-function rice mutant of this kinesin is not viable, the function was studied in tobacco BY-2 as heterologous system. OsDLK-GFP stably expressed in BY-2 cells decorates cortical microtubules, but also can shift into the nucleus of interphase cells. Because of this peculiar localisation, we coined the name Dual Localisation Kinesin (DLK). The nuclear import of this protein is strongly and reversibly promoted in response to cold. During mitosis, OsDLK is repartitioned between spindle and phragmoplast. Motility assays *in vitro* using show that OsDLK can convey mutual sliding of microtubules and moves at a velocity comparable to other class-XIV kinesins. When tobacco cells overexpressing OsDLK are synchronised, they exhibit a delayed entry into metaphase, while the later phases of mitosis are accelerated. The data are discussed in relation to additional functions of this kinesin type, beyond their transport along microtubules.

## Introduction

Plant cells show a distinct directionality (cell axis, cell polarity), which is guiding morphogenesis up to the organismic level. Both, microtubules and actin filaments, are endowed with an innate directionality as well, which is translated by molecular motors into a directionality of dynamic processes. One of the most striking peculiarities of plant directionality is the absence of microtubule minus end-directed cytoplasmic dynein motors in most Gymnosperms, and in all Angiosperms^1^. However, the minus end-directed kinesins^2,3^, generally referred to as class-XIV kinesins, have proliferated conspicuously, which is probably linked with the loss of flagella-driven motility that was progressively confined to the motile sperm cells (in Bryophytes, Pteridophytes, and early Gymnosperms), and, eventually, became dispensable by the development of a pollen tube. An interesting missing link is found in primitive gymnosperms, such as *Ginkgo* or *Cycas*, where the pollen tube bursts open only 50 µm before reaching the egg cell, releasing the flagellate spermatozoid^4^. The loss of flagellae was accompanied by a loss of centrioles as major microtubule-organizing centres, preceded by a progressive increase of acentriolar nucleation during mitosis. Instead, a novel microtubular structure, the phragmoplast, emerged in several lineages of the Green Algae and adopted the spatial organisation of the new cell plate (recently reviewed in^5^).

Since the functionality of the division spindle requires microtubular transport in both directions, the functions conveyed by dyneins in animal cells must be taken over by minus-end directed kinesins in plants. In fact, the class-XIV kinesins ATK1 (KatA) and ATK5 have been shown to bundle microtubules in the spindle mid-zone to generate inward forces to shorten the spindle length and focus spindle poles by gathering parallel microtubules towards the poles^6,7^. Of special interest are kinesin motors linked with phragmoplast and preprophase band, because these motors should reflect the cellular specificities of plant cell division. Indeed, the organisation of the phragmoplast seems to be linked with specific motors, such as the plant specific myosin motor myosin VIII^8^, the class-XII kinesins AtKinesins 12A, B^9^, the calcium-binding class-XIV kinesin KCBP^10^, and the class-XIV kinesin KCH^11^. Interestingly, some kinesins seem to be shared between phragmoplast and PPB. For instance, POK1 not only participates in the spatial control of cytokinesis^12^, but also, in concert with other proteins, tethers components required for later phragmoplast insertion at the site of the PPB^13^.

Similar to the phragmoplast, cortical microtubules (cMTs), the exclusive microtubule array in non-cycling plant cells, participate in the organisation of the cell wall^14^. Actually, it is this function, which led to the discovery of microtubules by Ledbetter and Porter^15^, and from the early days was proposed to be linked with transport along microtubules^16^. A class-IV kinesin, the KIF4 family member FRA1, is discussed as candidate for microtubular control of cellulose microfibril texture^17^. Interestingly, several kinesins are shared between phragmoplast and cortical array, including several class-XIV kinesins, such as ATK5^7^, or KCH^11^. This shared localisation might indicate that the machinery used to organise the new cell plate in dividing cells later has been recruited to organise also the cell wall in expanding cells. However, one should keep in mind that colocalisation with cortical microtubules does not necessarily mean that the respective kinesin is functional in organisation of cortical microtubules. For instance, although ATK5 accumulates at the plus ends of growing cMTs, the *atk5* mutant shows a normal organisation of cMT^7^.

Similar to the situation in animals, kinesins have progressively invaded other topological cellular functions in addition to mitotic chromosomal transport, such as the positioning of organelles, including premitotic nuclear migration^18^, transport of Golgi vesicles^19^, of mitochondria^20^, or light-induced chloroplast movement^21^.

A new and emerging topic is the link of such topological functions with signalling. The classical example is the kinesin-driven transport of synaptic vesicles in the axon - here, a directional transport function is used to sustain signalling. Likewise, non-translated mRNA for the transcription factor *oskar* driving gene expression required for abdominal development is located at the posterior pole of the *Drosophila* oocyte by virtue of a kinesin motor^22^. Signal-triggered, kinesin-dependent transport of a regulatory molecule can also be used to trigger specific responses in gene expression. For instance, in *Drosophila*, nuclear import of the CUBITUS INTERRUPTUS protein depends on the kinesin Costal2/Kif7, and regulates the hedgehog signalling pathway^23^. The surprising finding that the kinesin OsBC12/GDD1 not only binds to cortical microtubules, but can enter the nucleus and activate there a specific step of gibberellin biosynthesis, suggests that kinesin-dependent signalling is also a topic in the plant field^24^.

While in the dicot model *Arabidopsis* the closely related class-XIV kinesins ATK1 and ATK5 seem to localise both to the phragmoplast, the monocot model rice harbours only one homologue of these kinesins, leading to the question, whether this homologue (SwissProt accession number B8B6J5, GN = Os07g0105700) might represent a minimal system to fulfil the functions conveyed by ATK1 and ATK5. In this study, we characterized the molecular and cellular functions of this rice kinesin. However, the rice insertion mutant of OsDLK not only showed delayed seed germination, but even died in the early stage of seedling development. Thus, the function seemed to be essential, and we, therefore, used the approach to express this kinesin in tobacco BY-2 cells as heterologous system to address localisation and cellular functions. Using the recombinantly expressed full-length OsDLK, we showed by *in-vitro* sliding that it is a minus-end directed microtubule motor. A fusion with GFP decorates cortical microtubules, spindle, and phragmoplast. When the cell cycle was synchronised, the progression into metaphase was delayed in these overexpressor cells. Surprisingly, this protein was found to occur in two populations during interphase - one subpopulation was associated with cortical microtubules as observed in other class-XIV kinesins, the other population was localised inside the nucleus. This dual localisation was also confirmed by transient expression in other systems (*Arabidopsis* protoplasts, leaves of *Nicotiana benthamiana*, leaf sheath of rice). This partitioning of the protein to the nucleus could be induced by a chilling treatment and was reversible, when cells were returned to room temperature. Likewise, inhibition of nuclear export by Leptomycin B partitioned the protein to the nucleus. Since this kinesin was able to shuttle between two subcellular locations in a specific manner, we named this particular kinesin OsDLK for Dual Localisation Kinesin.

## Results

### Isolation and sequence analysis of OsDLK

**Figure 1:**
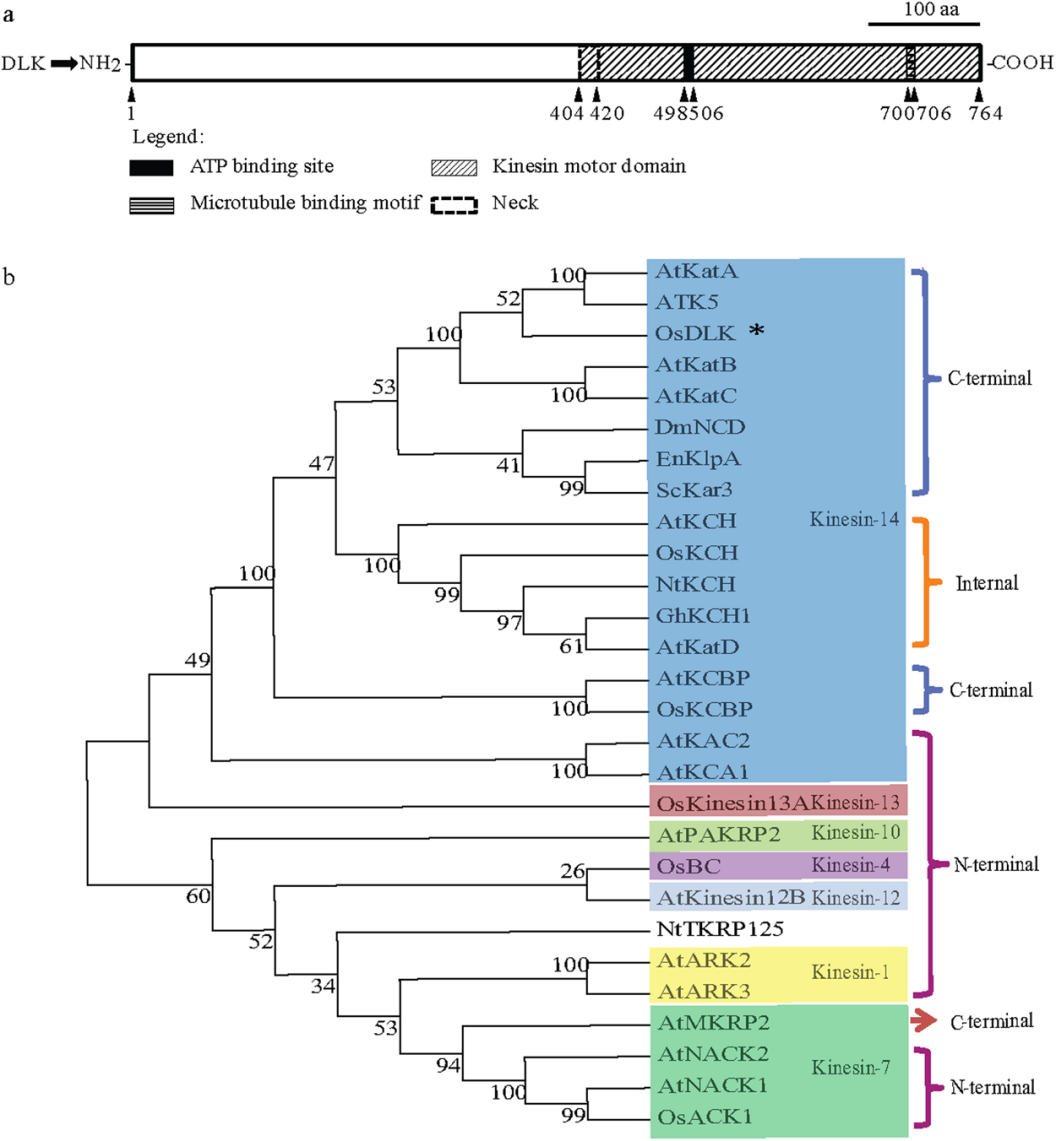
Sequence analysis of OsDLK from *Oryza sativa* L*. ssp. japonica*. (**a**) Predicted domains in the full-length protein (upper row). A putative kinesin motor domain is found in the C-terminal region and includes a conserved neck region, and ATP, as well as microtubule-binding sites. (**b**) Phylogenetic relationship of OsDLK (marked by an asterisk) with other members of the kinesin-14 family and selected members from other several kinesin subgroups from different animal and plants.

A putatively full-length cDNA for rice DLK (Dual Located Kinesin) was isolated by RT-PCR. The obtained amino-acid sequence coding for the OsDLK protein is identical to the sequence published for the rice reference genome (UniProtKB/Swiss-Prot accession no. B8B6J5). The putative kinesin is predicted to harbour a highly conserved kinesin motor domain (aa 404–764) at the C-terminal region, including an ATP-binding consensus (aa 498–506), a putative microtubule binding site (aa 700–706)^25^ (Fig. 1a), and a coiled-coil stalk (aa 109–422) for protein dimerization^26^. A 14-amino-acid neck-linker region (aa 405–418) directly upstream of the catalytic core comprises the consensus neck motif found among kinesins that move towards the minus end of microtubules^27^. Specifically, this region contains two critical amino acids known to be crucial for kinesin minus-end directed movement (Supplementary Fig. S1). Thus, OsDLK displays all sequence motives indicative of a microtubule minus end-directed motor. Interestingly, three putative Nuclear Localization Signal are predicted by algorithms such as the http://nls-mapper.iab.keio.ac.jp/cgi-bin/NLS_Mapper. While one motif of 11 amino acids length (aa 401–411) overlaps with the neck-linker domain, there are two additional putative bi-partite sites with long linkers at positions 64–93 and 207–237.

OsDLK shows clear homology with other kinesin-14 sequences known from other organisms (Supplementary Fig. S1). For instance, the N-terminus of the *Arabidopsis* kinesins ATK1 and ATK5 (with mutual amino-acid identities of 75.5%), exhibit 38.2% and 40.6% amino-acid similarity to OsDLK, respectively. In the motor domains, both ATK1 and ATK5^7,28^ showed around 75% amino-acid identity to OsDLK. Both ATK1 and ATK5 are C-terminally localized kinesins with a coiled-coil stalk in the middle of the protein. A phylogenetic tree (Fig. 1b) placed OsDLK (marked by an asterisk) clearly into the C-terminally class-XIV kinesins with a close relationship to ATK5 and ATK1.

*In-silico* data on expression patterns obtained from the *Genevestigator* microarray database^29,30^ indicate an overall high expression in all tested tissues of rice as well as through all developmental stages (Supplementary Fig. S2).

### Dual localisation of OsDLK during interphase

In order to gain insight into the unknown functions of OsDLK during the cell cycle, two constructs (OsDLK-GFP and OsDLK-RFP) were generated for stable and transient expression in tobacco BY-2 cells, respectively, whereby a full-length OsDLK cDNA (2295 bp) was fused upstream of the green fluorescent protein (GFP) or red fluorescent protein (RFP).

**Figure 2:**
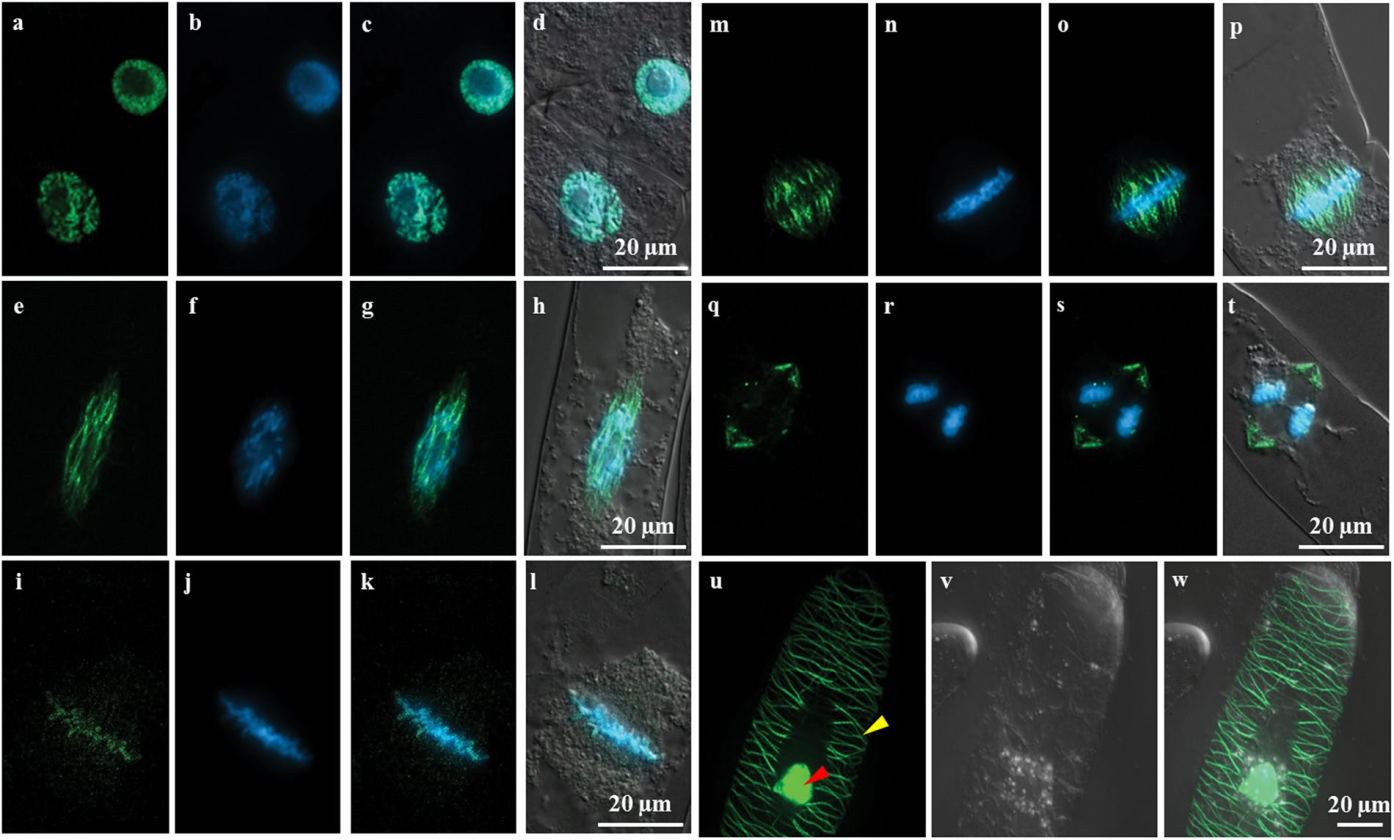
Subcellular localisation of OsDLK-GFP upon heterologous expression tobacco (*Nicotiana tabacum)* BY-2 cells. (**a**–**t**) Cells in subsequent stages of mitosis upon dual visualisation of full-length OsDLK (GFP signal) and DNA (Hoechst 33258). (**a**–**d**) preprophase. (**e**–**h**) prophase. (**i**–**l**) metaphase. (**m**–**p**) Transition metaphase to anaphase. (**q**–**t**) Transition anaphase to telophase. The GFP signal indicative of OsDLK is shown in a, e, i, m and q; the Hoechst 33258 signal indicative of DNA is shown in b, f, j, n and r; both GFP and Hoechst signal channels are merged in c, g, k, o and s; d, h, l p and t show the merge of all the channels. (**u**–**w**) Dual localisation of full-length OsDLK in fusion with GFP during interphase. GFP signal (**u**), differential interference contrast (DIC) (**v**) and merged images (**w**). Signals of OsDLK-GFP in nucleus and cortex are shown with red and yellow arrows, respectively. Scale bars: 20 μm.

**Figure 3:**
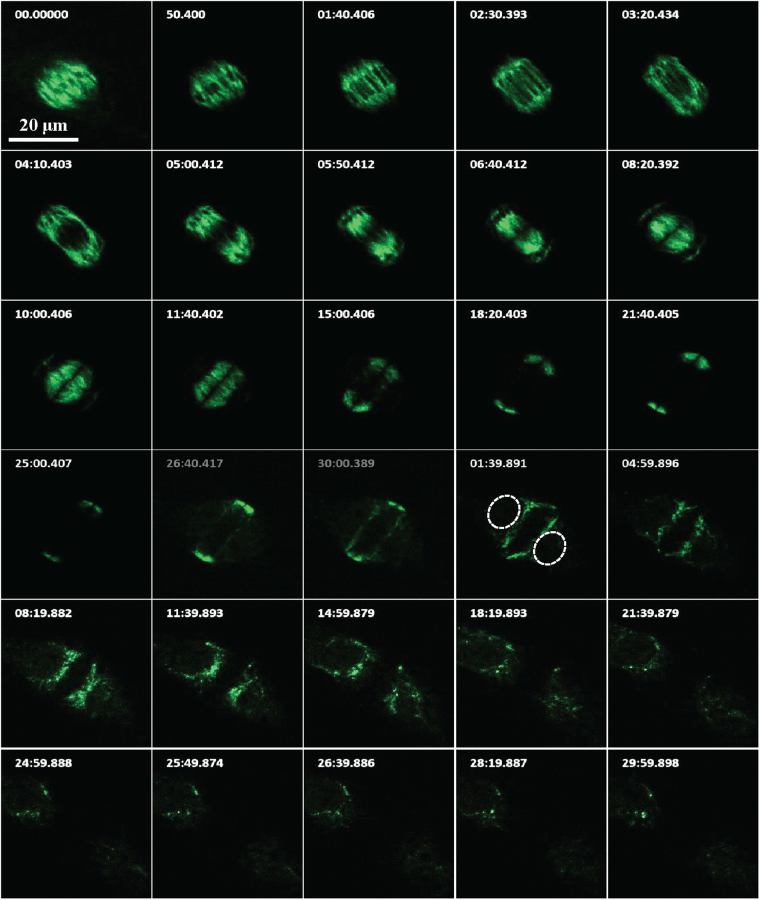
Time-lapse series of OsDLK-GFP localisation during the later phases mitosis and during cytokinesis (see also Supplementary Movie S1), time unit: s. The nucleus is marked with white dotted line. Scale bars: 20 μm.

When the subcellular localisation of OsDLK-GFP was followed through the cell cycle (Fig. 2), the fluorescent signal was seen to undergo a dynamic reorganisation: During prophase, the signal in the cell cortex disappeared completely, and the intranuclear signal appeared agglomerated (Fig. 2a–d) and was later replaced by a mesh-like structure wrapping the nucleus (Fig. 2e–h). During early metaphase, OsDLK-GFP was found in form of (relatively scarce) beads on a string distally from the metaphase plate, along with agglomerations in the metaphase plate, mostly proximally of the chromosomes (Fig. 2i–l). During late metaphase (Fig. 2m–p), the signal distal to the metaphase plate had increased into clear and continuous fibres, whereas the signal in the metaphase equator had almost vanished. When anaphase was completed, OsDLK-GFP returned to the equatorial region (Fig. 2q–t) localising to give rise to the phragmoplast, i.e. to the microtubular array which deposits cell plate material as it expands outward, and therefore, similar to the cortical microtubules that form later, associates with the cell wall. While OsDLK-GFP was found in all arrays of microtubules, it was not seen to label all microtubules of these arrays, but was concentrated at specific flanks of the structure, for instance in the late anaphase spindle (Fig. 3).

**Figure 4:**
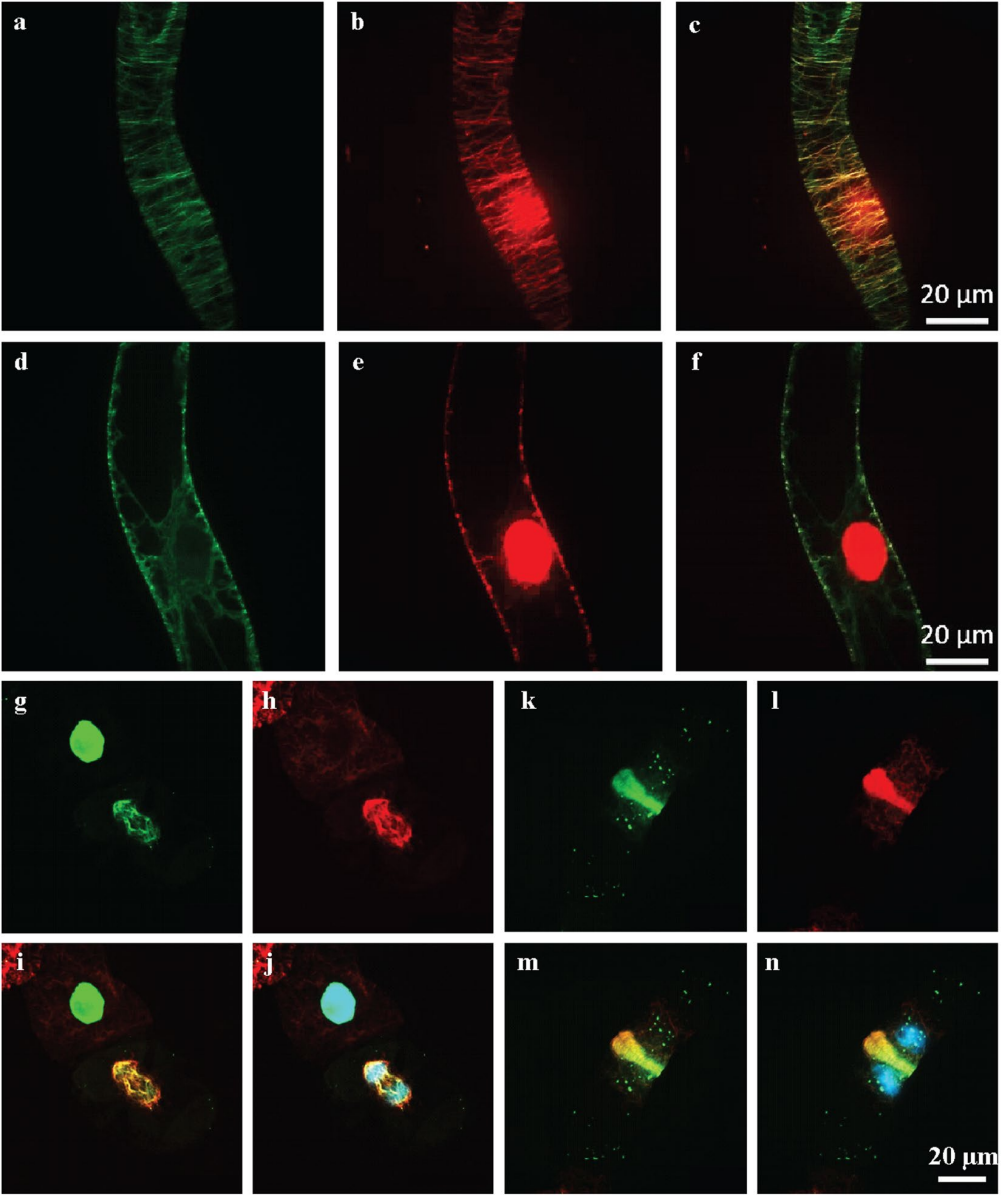
Co-localisation of OsDLK fusions with fluorescent proteins and microtubules upon heterologous expression in tobacco BY-2 cells. (**a**–**f**) Cortical and central confocal section of a cell transiently transform with OsDLK-RFP and GFP-pCambiaTuB6 showing the colocalisation of OsDLK (**b**) with cMTs (**a**) in periphery, and the intranuclear localisation of OsDLK-RFP (**e**) while GFP-Tub6 (**d**) localized in radial MTs tethering the nucleus. Merged signals are shown in c and f. (**g**–**n**) Triple staining of OsDLK-GFP, microtubules visualised by immunofluorescence with rhodamine, and DNA visualised by Hoechst 33258 of representative cells in late anaphase (**g**–**j**) and telophase (**k**–**n**). The OsDLK-GFP signal is shown in g and k, the microtubule signal in h and l, the merge of these signals in i and m, the merge of all three signals in j and n. Scale bars: 20 μm.

**Figure 5:**
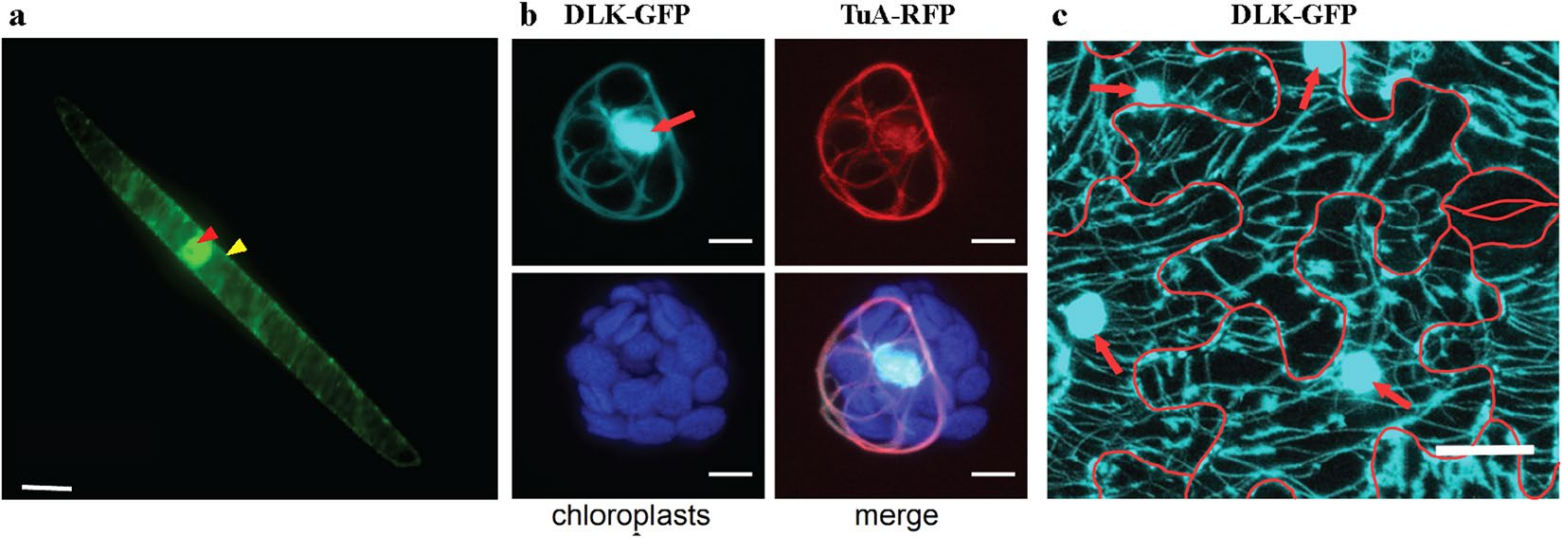
Dual localisation of full-length OsDLK-GFP in different plant models. (**a**) Dual localisation of full-length OsDLK-GFP in interphase cells of the rice leaf sheath. Nuclear and cortical OsDLK-GFP signals are indicated by red and yellow arrows, respectively. Scale bar: 20 µm. (**b**) Co-localisation of OsDLK-GFP and microtubules in *Arabidopsis thaliana* protoplasts. Representative maximum intensity projections of fluorescent image stacks showing transient expression of OsDLK-GFP (cyan), stable expression of the microtubule marker TuA-RFP (red), and chloroplast autofluorescence (blue) in an *Arabidopsis thaliana* mesophyll protoplast. Scale bar: 5 µm. (**c**) Representative maximum intensity projection of fluorescent image stacks showing transient expression of OsDLK-GFP (cyan) in *Nicotiana benthamiana* leaf pavement cells. Red arrows show the OsDLK-GFP localisation in the nuclei. Cell borders are indicated by red lines generated from bright field images taken at the same field of view. Scale bar: 25 µm.

During interphase, surprisingly, OsDLK-GFP was localised in two populations: On the one hand, OsDLK-GFP was continuously decorating cortical microtubules (Figs 2u–w, 4a–c). Simultaneously, intensive fluorescent signals were found inside the nucleus (Figs 2u–w, 4d–f). The dual localisation of OsDLK in cell cortex and nucleus was therefore validated in additional systems: the epidermal cells of the rice leaf sheath as homologous system (Fig. 5a), and two further experimental models, *Arabidopsis thaliana* mesophyll protoplasts, and pavement cells of *Nicotiana benthamiana* leaves as additional heterologous systems (Fig. 5b,c).

To get more insight into these complex migrations of OsDLK-GFP during the later phase of mitosis, detailed time-lapse series were recorded (Fig. 3, Supplementary Movie S1). These series show how OsDLK-GFP at the final stage of metaphase is organised in rod-like structures at the proximal edge of the metaphase plate that are aligned poleward. Then, within 3 min, the signal moves towards the spindle poles and contracts in two helmet-like clusters just beneath a terminal, smaller cluster. Subsequently, the entire structure shortens rapidly, such that 5 min later the two helmets have reached the equator again lining from two sides a dark zone that probably corresponds to the newly emerging cell plate. During this contraction process, the first strongly aggregated bundles in the helmet detach into finer fibres that probably represent the microtubules of the phragmoplast. During expansion of this *bona-fide* phragmoplast, OsDLK-GFP remains over the next 15 min at the site, where phragmoplast microtubules are presumed to be. That OsDLK-GFP is indeed decorating phragmoplast microtubules, can been shown by visualising microtubules by immunofluorescence (Fig. 4k–n). Afterwards, the signal starts to appear at the nuclear envelopes of the newly formed daughter nuclei, and first concentrates at the trailing edge of the nuclei (that move apart from the cell plate). Eventually, when the daughter nuclei have reached their position in the symmetry planes of the newly formed cells, this gradient is progressively levelled out.

To test, whether OsDLK was really associated with microtubules, we used two approaches - transient co-transformation of OsDLK-RFP and the microtubule marker TuB6-GFP, as well as immune-labelling of microtubules in cells expressing OsDLK-GFP. We found that OsDLK-RFP decorated the GFP-labelled cortical microtubules (Fig. 4a–c), whereas the nucleus of the same cells harboured the RFP signal indicative of OsDLK, but no microtubular signal (Fig. 4d–f). This uncoupling of the two signals in the nucleus could also be confirmed using TRITC-based immunostaining of microtubules (Fig. 4g–j). Conversely, immune-labelling of the phragmoplast (Fig. 4k–n) showed a tight colocalisation of OsDLK-GFP and microtubules, whereas the interior of the newly formed daughter nuclei was not labelled. Thus, OsDLK-GFP co-localised with the wall associated arrays of microtubules (cortical microtubules, phragmoplast). However, during interphase, it can occur in a second form that resides inside the nucleus and seems to be dissociated from microtubules. The fact that the intranuclear signal depends on the cell cycle (present in interphase, absent in telophase) argues against a scenario, where GFP or RFP are cleaved from DLK fusions, also because a cleaved label should distribute equally through cytoplasmic strands, which is not seen.

**Figure 6:**
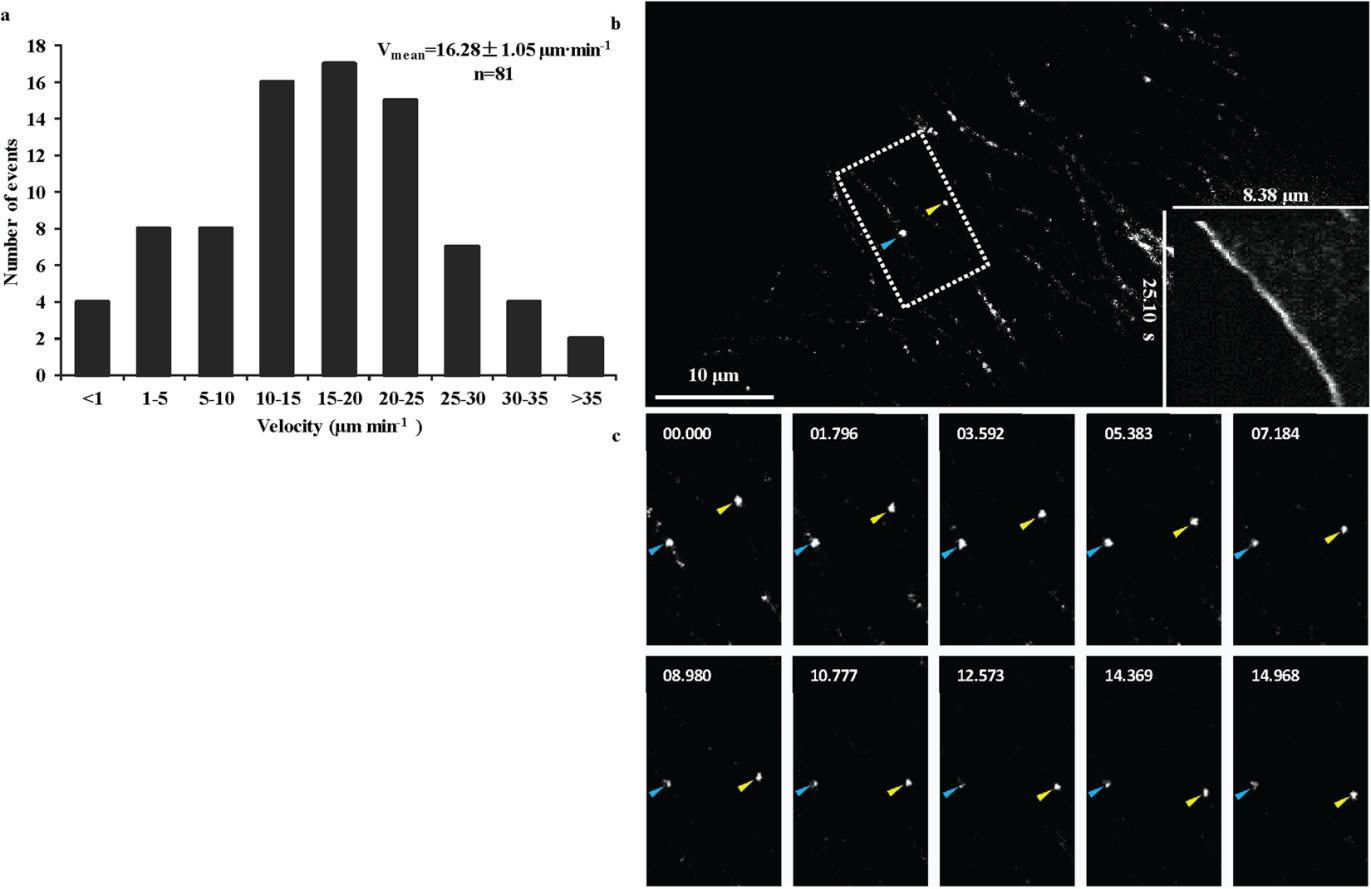
Time-lapse studies on the dynamic behaviour of different OsDLK subpopulations in stably transformed OsDLK-GFP BY-2 cells. (**a**) Velocity distribution of OsDLK-GFP moving on cortical microtubules (cMTs) with an average velocity 16.28 ± 1.05 μm·min^−1^ (x ± SE; n = 81). (**b**) Representative kymograph experiment showing kinesin movement in an interphase BY-2 cell. The kymograph shown in the bottom right corner represents the OsDLK-GFP signal marked by a yellow arrowhead. The left bar indicates the time, and the top bar indicates the distance. (**c**) Time-lapse series showing in detail the dynamic behaviour of the OsDLK-GFP highlighted with blue and yellow arrowhead (see Supplementary Movie S2). Time unit: s, Scale bars: 10 μm.

The kymograph data revealed that OsDLK-GFP is a dynamic motor, moving with an average *in-vivo* speed of 16.28 ± 1.05 μm.min^−1^ on cMTs (Fig. 6a–c, Supplementary Movie S2). This speed exceeds that of NtKCH, a different member of the class-XIV kinesins (Table 1). However, there exist members of other kinesin families (such as RnKHC, a class-I kinesin) that are much faster.

**Table 1:** Velocity of OsDLK-GFP compared to velocities of other kinesins from plant and animal species and the conventional KHC from *Drosophila melanogaster* and *Rattus norvegicus*.

Kinesin	Velocity (μm·min^−1^)	(Predicted) Directionality	Position	Reference
OsDLK	16.28 ± 1.1	−	*In vivo*	*This research*
	5.2 ± 1.1	−	*In vitro*	
DmNCD	8–12	−	*In vitro*	^64^
AtKatA	9.6 ± 5.2	−	*In vitro*	^28^
OsKCH1	5.4 ± 2.3	−	*In vitro*	^65^
NtKCH	3.26 ± 0.1	−	*In vivo*	^11^
ATK5	6.30 ± 1.4	−	*In vitro*	^7^
ScKar3	1.30 ± 0.1	−	*In vitro*	^66^
AtKCBP	10.0 ± 0.4	−	*In vitro*	^67^
RnKHC	46.8 ± 6.6	+	*In vivo*	^68^
RnKin430	52.4 ± 1.5	+	*In vitro*	^69^
DmKHC	45.6 ± 6.0	+	*In vitro*	^70^

### Overexpression of OsDLK-GFP prolongs mitosis

**Figure 7:**
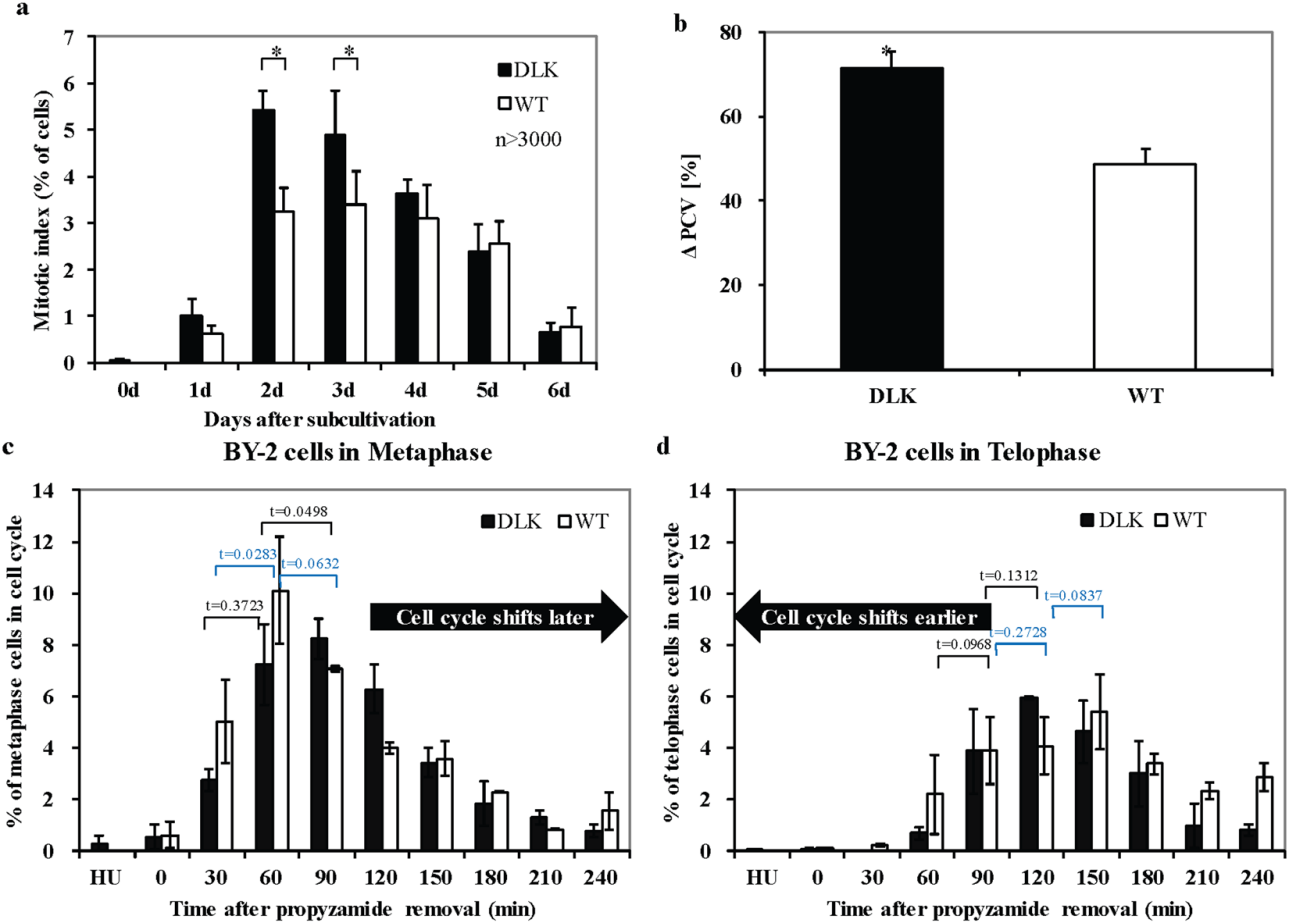
Phenotype detection of OsDLK-GFP overexpressor. (**a**) Stimulation of mitotic index in OsDLK-GFP BY2 compared to non-transformed BY-2 WT. More than a total of 3000 cells per time point and sample were collected cumulatively from three independent experimental series. Asterisk (*) indicate significant differences between the cell lines at P < 0.05 as evaluated by a t test for unpaired data. Error bars represent the standard error of triplicate measurements. (**b**) Increased packed cell volume in the OsDLK-GFP overexpressor compared to the non-transformed BY-2 WT during 4 to 6 days after subcultivation. *Significant difference between the cell lines at P < 0.05 as evaluated by a t test for unpaired data. Error bars represent the standard error of triplicate measurements. (**c,d**) OsDLK-GFP cells (black bars) progress through mitosis with a different temporal pattern compared to the non-transformed wildtype (white bars). (**c**) Frequency of metaphase cells over the time after release from propyzamide. (**d**) Frequency of telophase cells over the time after release from propyzamide. Error bars represent the standard error of biological triplicates comprising a population of 3000 cells per data point.

To get insight into the cellular function of OsDLK-GFP, mitotic index and cell expansion were followed in comparison with the non-transformed BY-2 cell line. The mitotic index of the expressor was significantly higher during the 4 days after subcultivation, especially at the second and third day (Fig. 7a). Since the doubling time of the transgenic cell line was determined to be similar to WT (Supplementary Fig. S3a), the increase in mitotic index was not caused by a higher frequency of cells entering mitosis, but by a longer passage through the mitotic phase.

However, during the cell expansion (during 4 day to 7 day cultivation), the packed cell volume (PCV) of the OsDLK-GFP overexpressor was significantly increased compared to the WT cell line (Fig. 7b), which compensated the decrease during the first 3 days of cultivation. So that the PCV exhibited no difference after 6 days of cultivation. In order to determine whether this compensation was due to cell expansion or cell proliferation, cell length and width were determined. The cells of OsDLK-GFP overexpressor were more elongated than the WT cells, while the increment of cell width was reduced. Thus, overexpression of OsDLK-GFP slowed mitotic activity during the proliferation phase of the culture while delaying cell elongation. This, along with a more pronounced elongation during the expansion phase of the culture resulted in a similar final packed cell volume.

### Overexpression of OsDLK-GFP delays the transition into metaphase

The fact that the OsDLK-GFP overexpressor exhibited a higher mitotic index, but a similar length of the cell cycle indicates that the lines differ with respect to the duration of mitosis. We, therefore, monitored the temporal progression through mitosis in synchronized BY-2 cells. Cells were treated first with the ribonucleotide reductase inhibitor hydroxyurea (which arrests the cells in S-phase) and then with the reversible anti-microtubular inhibitor propyzamide (which arrests the cells in prophase). Following the treatment with hydroxyurea, the mitotic index was 0%, indicative of full suppression of cell cycle progression into the M-phase. Upon release from propyzamide, the non-transformed cell line had a more efficient synchronisation with a higher MI (70%) while it was lower (60%) in the OsDLK-GFP overexpressor line (Supplementary Fig. S4a,b).

The frequency of the individual mitotic phases was followed over time (Supplementary Fig. S4c,d). Whereas the metaphase peak in the OsDLK-GFP line was seen 90 min after removal of propyzamide, which was half an hour later than in the WT (Fig. 7c), the telophase peak of OsDLK-GFP cells occurred 30 min earlier (Fig. 7d). These time courses report that, in the OsDLK-GFP overexpressor, the mitotic phases preceding metaphase are prolonged, whereas the mitotic phases following metaphase are accelerated. These results are supported by time courses of nuclear positioning recorded over the cultivation cycle (Supplementary Fig. S5). Here, the initial premitotic migration of the nucleus from the lateral wall to the cell centre was delayed in the OsDLK overexpressor.

### Nuclear import of OsDLK-GFP in response to cold stress

**Figure 8:**
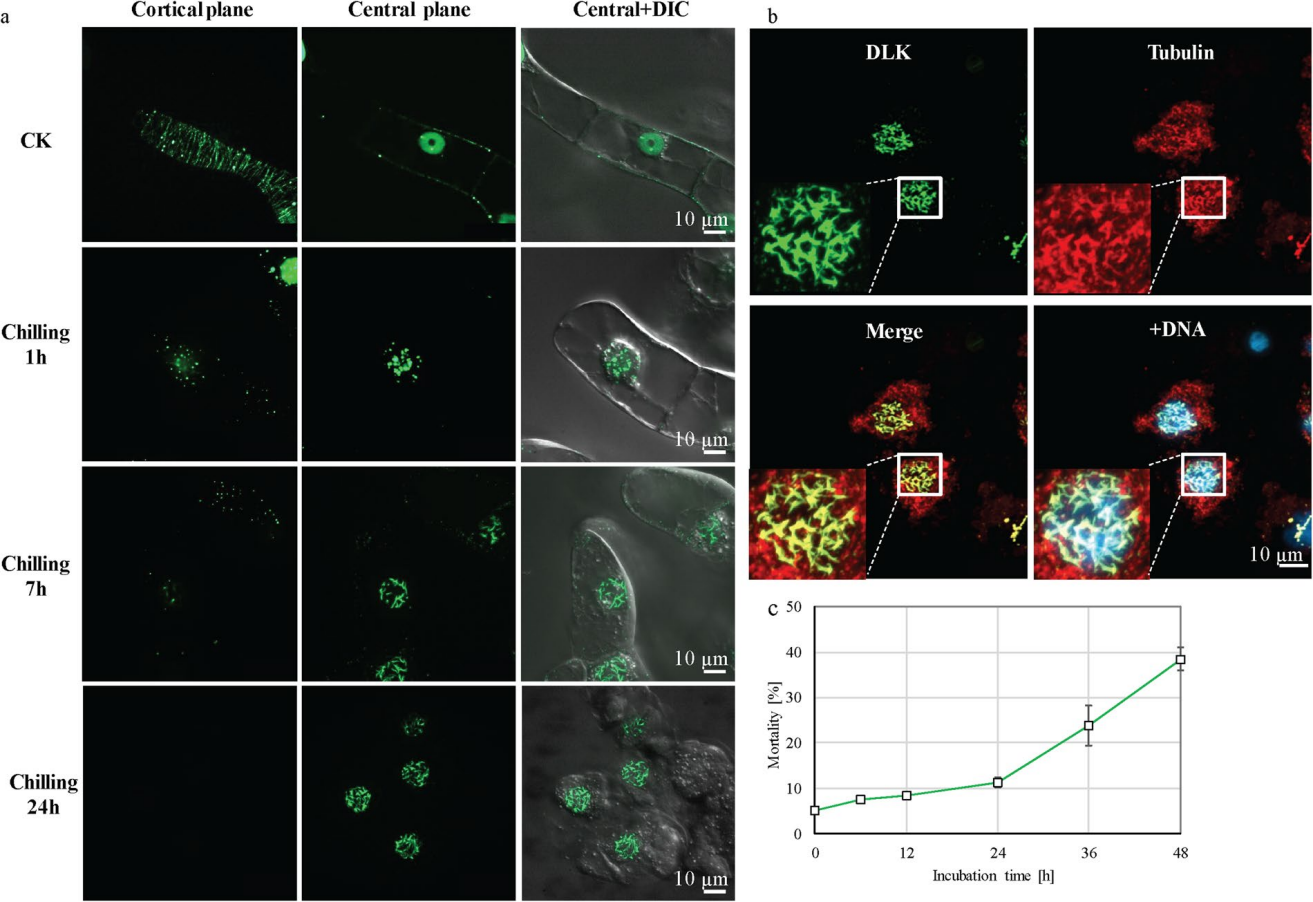
OsDLK-GFP enters the nucleus in response to cold stress. (**a**) Progressive nuclear import of OsDLK-GFP with increasing time of cold treatment. Confocal sections collected from the cortical and nuclear planes are shown either for the GFP signal alone or merged with the differential interference contrast image to show the topology. CK represent cells cultivated at 25 °C serving as negative control. (**b**) Representative cells challenged by cold for 7 hours and triple stained for microtubules (immunofluorescence using a TRITC conjugated antibody), OsDLK-GFP, a merge of both signals, and DNA visualised by Höchst 33258. The white square has been magnified to show details of colocalisation (arrow). Scale bars: 10 μm. (**c**) Time course of non-transformed BY-2 cells mortality after treatment with ice water.

To determine, whether the two interphase population of OsDLK-GFP (at cMTs and inside the nucleus) can be interconverted, the OsDLK-GFP cells were followed during their response to cold stress, since cold treatment can induce a nuclear import of tobacco tubulin^31^. With progressive time of cold treatment, the GFP signal indicative of OsDLK disintegrated into punctate residual signals in the cell cortex; simultaneously the signal accumulated inside the nucleus, as well in a punctate manner (Fig. 8a). This response was rapid and already clearly manifest after 1 h of cold treatment. With progressive cold treatment, the intranuclear signal organised in rods and filaments, evident from 7 hours after the onset of the treatment. After 24 hours, the cortical signal had vanished completely, whereas the filamentous organisation of the intranuclear signal was fully developed. To get insight into the nature of these filaments, immunostaining of microtubules was carried out in the background of OsDLK-GFP cells following cold treatment for 7 hours (Fig. 8b). Tubulin was seen in and around the nucleus in form of punctate or sometimes rod-shaped structures, whereas the cortical microtubules were not detectable. The signal for OsDLK-GFP was exclusively observed inside of the nucleus. Here, the filamentous or rod-shaped structures visualised by OsDLK-GFP tightly overlapped with the tubulin signal and also with the chromatin (Fig. 8b, insets, white frame), whereas in the cytoplasm around the nucleus (delineated by the absence of DNA), the tubulin signal was not accompanied by OsDLK-GFP signal. The viability of non-transformed BY-2 cells in ice water was followed by the Evans Blue dye exclusion assay (Fig. 8c). In the time range till 24 h, the mortality is only around 10%. Only for longer treatments mortality increases significantly. Thus, during the time intervals used for cold treatments inducing nuclear localisation of DLK cells are almost fully viable. To test, whether the nuclear import of OsDLK-GFP was reversible, we conducted a recovery experiment, where cells were subjected to cold treatment for 7 h to induce complete dismantling of the cortical signal. Then, the cells were returned to 25 °C for recovery. During recovery, the rod-like structures in the nucleus disassembled, such that the signal was spread more or less evenly over the entire karyoplasm (Supplementary Fig. S6). At the same time, punctate signals appeared in the cytoplasm around the nucleus that were aligned in transverse orientation like beads on a string. Thus, the nuclear import and the intranuclear filamentous organisation of OsDLK-GFP were reversible.

### Leptomycin B causes accumulation of OsDLK-GFP in the nucleus

**Figure 9:**
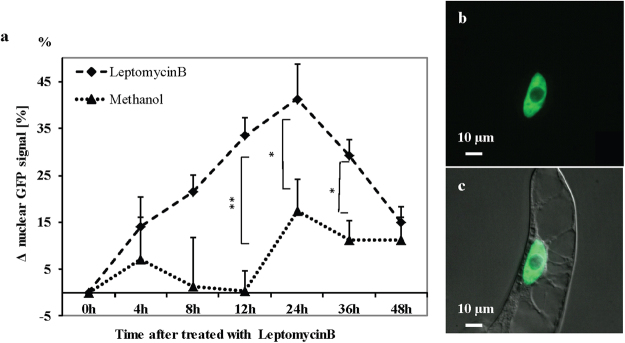
Effect of Leptomycin B, a specific inhibitor of nuclear export on localisation of OsDLK-GFP. (**a**) Time course of intranuclear signal increment in response to 200 nM LeptomycinB compared to the solvent control (the same volume of 70% methanol, corresponding to a final concentration of 1.37% MetOH in the assay as control). At least a total of 20 cells per time point and sample were collected in each experimental series. The results were tested for significance using Student’s t-test at 95% and 99% confidence level, labled with asterisks. Error bars represent the standard error of triplicate measurements. (**b**,**c**) Representative image of an OsDLK-GFP cell after 3 days of treatment with 200 nM Leptomycin B. GFP signal shown in (**b**), overlay with differential interference contrast shown in (**c**). Scale bars: 10 μm.

To get insight into the mechanism responsible for the cold-induced accumulation of OsDLK-GFP in the nucleus, we treated OsDLK-GFP cells with Leptomycin B (a specific inhibitor of nuclear export^32^), but in the absence of cold stress, i.e., at 25 °C. We quantified the proportion of GFP signal located inside the nucleus and followed this parameter over time of Leptomycin B treatment (Fig. 9a). In the OsDLK-GFP cells, the proportion of intranuclear GFP increased by around 40% of the initial value over 24 hours after onset of the treatment, and then dropped back to an intermediate level during the following day. The solvent control did not show this sharp increase, although it should be noted that values increased as well, however only by 15% at 24 h and then levelled off at below 10% for longer incubation. This increase of the intranuclear signal was linked with the loss of the cortical signal (Fig. 9b,c). These results indicate that the intranuclear OsDLK-GFP signal results from a dynamic equilibrium established by import and export. This cycling takes place also at normal temperature. On the assumption of around 50% of the signal being located in the nucleus under steady-state conditions (Fig. 9a), and the increase of the intranuclear signal to around 75% within 24 h of Leptomycin B treatment, it can be estimated that, at 25 °C, roughly half of the intranuclear population is turned over within one day. Compared to the accumulation of signal observed under cold stress, the entry of DLK under room temperature must be much slower (almost by one order of magnitude).

### OsDLK is capable of MT sliding *in vitro*

**Figure 10:**
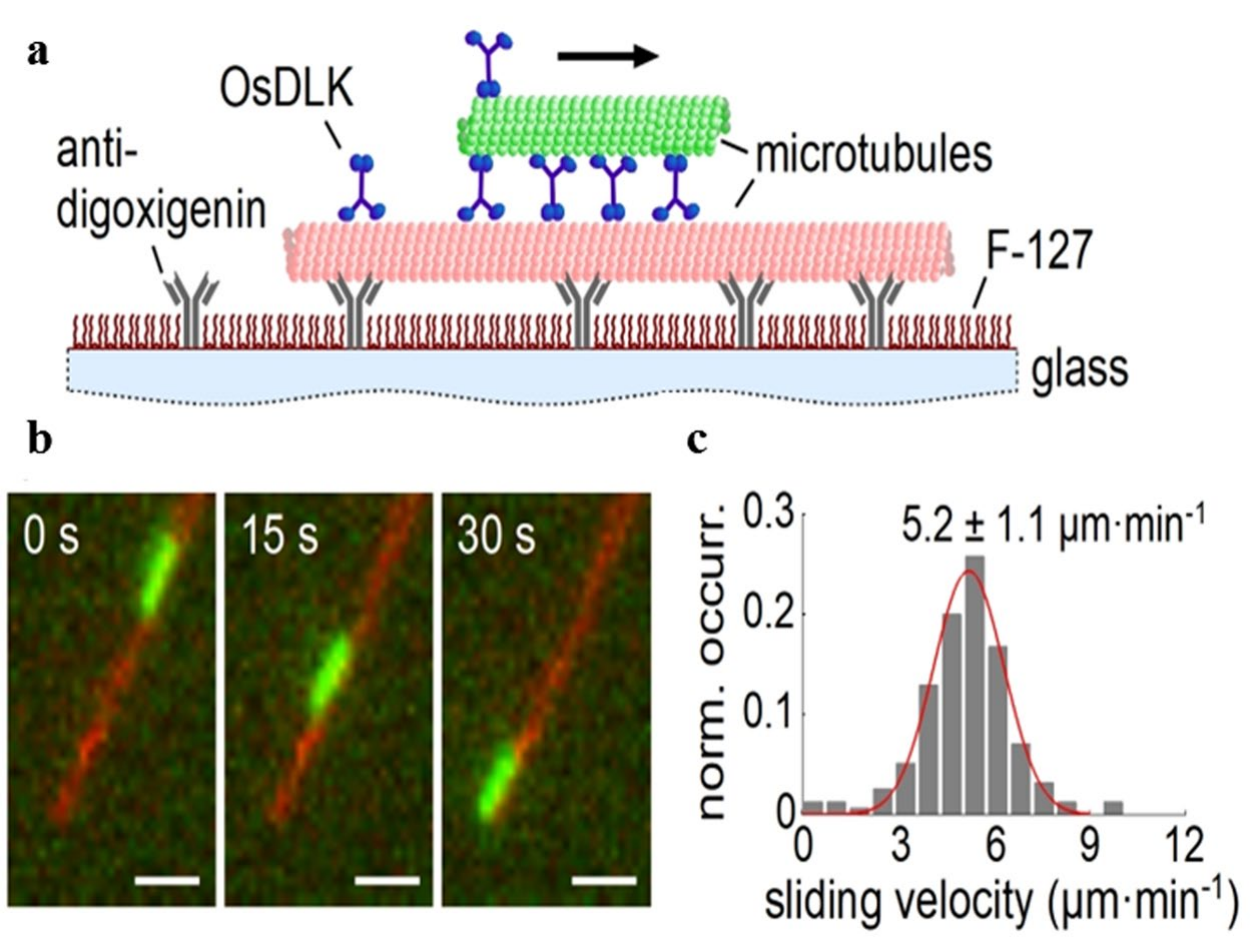
OsDLK is a microtubule minus-end-directed motor along microtubules. (**a**) Schematic representation of the microtubule sliding assay using recombinant OsDLK (see Methods for details). (**b**) Fluorescence micrographs of a cargo microtubule (green) being transported by OsDLK along a surface-bound template microtubule (red) at different points in time. Scale bar = 1 µm. (**c**) Histogram of the point-to-point sliding velocities. A Gaussian fit of the histogram delivers the sliding velocity v = 5.2 ± 1.1 μm·min^−1^ (mean ± SD, N = 155).

In order to study the interaction of OsDLK with microtubules, we performed microtubule sliding assays, where motors could interact simultaneously with surface-immobilized and free microtubules in the presence of ATP (Fig. 10a). We found that OsDLK actively transports microtubules along each other in a unidirectional manner *in vitro* (Fig. 10b) with a velocity of v = 5.2 ± 1.1 μm·min^−1^ (mean ± SD, *N* = 155, Fig. 10c), which is comparable to the other members of the class-XIV family (Table 1).

## Discussion

The current work deals with the functional characterisation of a class-XIV kinesin from rice, which is homologous to the *Arabidopsis* kinesins ATK1 and ATK5. While this rice homologue shares several molecular and cellular features with its *Arabidopsis* counterparts, moves towards the minus end of microtubules in a dynamic fashion, and shows a similar dynamic relocalisation during mitosis, it shows a specific difference during interphase: A part of this rice kinesin decorates cortical microtubules, while the other part is found in the nucleus. In response to cold treatment (eliminating cortical microtubules), the intranuclear population increased and formed rod-shaped and reticulate structures. Likewise, inhibition of nuclear export by Leptomycin B increased the abundance of intranuclear kinesin, such that the name Dual Localisation Kinesin (DLK) was coined for this protein.

Based on sequence homologies in their motor domains, kinesins are classified into 14 sub-families^33^. In land plants, the class-XIV kinesins have strongly expanded, and many of them differ greatly in structure and function from their animal counterparts. In this study, we investigate a rice homologue, of two similar *Arabidopsis* class-XIV kinesins, ATK1 and ATK5^6,7^ with overlapping, but not identical function. This rice homologue, designated as OsDLK (for Dual Localisation Kinesin) shows typical structural features of class-XIV kinesins, such as a highly conserved motor domain at the C-terminus with an ATP binding motif, a long stalk region in the middle, and a tail at the N-terminus^34^. The amino-acid signature in the neck region (Supplementary Fig. S1) predicts that OsDLK is moving towards microtubular minus-ends. The motor velocity *in vitro* is comparable to other class-XIV kinesins (around 5 µm.min^−1^, Fig. 10), while the motility along cortical MTs *in vivo* is considerably higher (16 µm.min^−1^, Table 1), excelling the velocity of its *Arabidopsis* counterpart ATK5 almost threefold^7^. Whether this high velocity *in vivo* is caused by motor clusters, or by cotransport with growing or shrinking MT ends, remains to be elucidated, for instance by means of single-molecule tracking *in vitro*. However, except this elevated velocity (which is not unusual if compared with other kinesins), the molecular features of OsDLK are that of a mostly non-conspicuous member of the class-XIV kinesin family.

As the rice mutant was not viable beyond early seedling development (Supplementary Fig. S7), OsDLK was assumed to convey essential functions, such as the cell cycle. Thus, the function was addressed in tobacco BY-2, as classical model, where the cell can be synchronized efficiently. To avoid that individual insertion events bias the readout, and to overcome potential impact from somatoclonal variation which is always an issue in cell culture systems, we pooled the different calli during transformation. Thus, any significant phenotype averages over a population of different clones, where the transgene has been inserted in different sites. As fundamental process in plant development, cell division is a major target for kinesins. For instance, in *Arabidopsis*, more than one third of kinesins have been found to participate in mitosis^35^. In a synchronized cell culture of *Arabidopsis*, 7 class XIV- kinesins were upregulated during re-entry into the cell cycle^36^, suggesting a core role in mitosis. Consistent with this, transgenic BY-2 cells overexpressing OsDLK showed characteristic alterations of proliferation with a higher frequency seen in mitosis (Fig. 7a,b). At the same time, the doubling time of the culture was not altered (Supplementary Fig. S3a), indicative of a prolonged progression through mitosis. In fact, synchronisation experiments showed that the OsDLK line required more time to pass metaphase, while telophase was passed more rapidly (Fig. 7c,d). During the subsequent expansion phase of the culture, The transgenic cells passed more rapidly from proliferation to expansion, as indicated by accelerated nuclear migration from the cell centre towards the periphery after proliferation had ceased (Supplementary Fig. S5), accompanied by a (slight) promotion of cell elongation (Fig. S3b,c). Interestingly, the pattern differs from that seen for overexpression of a different class-XIV kinesin, OsKCH. Here, the exactly opposite pattern had been observed, where the overexpressors show stimulated cell elongation but delayed mitosis during the first 3 days after subcultivation^18^. Thus, different members of this kinesin class seem to convey different and specific functions.

When we followed the dynamic redistribution of OsDLK, we observed a pattern that is known from other class-XIV kinesins: In yeast and human cells, class-XIV kinesins (antagonised by class-V kinesins) can regulate microtubule nucleation through the γ-TuRC complex and are conserved elements during formation and function of the spindle apparatus^37^. Typically, class-XIV kinesins bind to the midzone of spindle, thus contributing to the straightening of the spindle axis and the shortening of the spindle. Consistently with this rule, OsDLK accumulated in the metaphase plate and then redistributed into clear fibres distal to the metaphase (Fig. 2i–p). A second feature of class-XIV kinesins is the bundling of parallel MTs to focus the spindle poles. Conversely, OsDLK accumulated at the minus ends of parallel MTs at the spindle pole during anaphase (Supplementary Movie S1), as shown for ncd^38^. Overall, the localisation pattern of OsDLK resembles that of its *Arabidopsis* homologues, ATK1 and ATK5, both of which are observed at spindle poles and midzone^6,7^. This indicates that ATK1/ATK5 and OsDLK may have similar functions in mitosis. Whether OsDLK contributes to microtubule bundling during organisation of the PPB, as had been shown for KCBP^39,40^, remains to be elucidated. The fact that binding of OsDLK to the PPB was not observed during this study, along with the fact that overexpression of OsDLK in BY-2 cells did not lead to abnormal spindles, would be more consistent with a function of OsDLK that differs from that of KCBP.

While the mitotic localisation of OsDLK was consistent with the findings on its *Arabidopsis* homologues ATK1 and ATK5, its subcellular localisation during interphase was unexpected. During interphase, OsDLK was found in two populations, one associated with cortical microtubules, the other inside the nucleus. Moreover, this localisation was dynamic and regulated in response to cold (in a reversible manner). Thus, this kinesin was repartitioned from the cortical cytoplasm into the nucleus, and even in the absence of cold the intranuclear population of DLK was constantly cycled between cytoplasm and nucleoplasm, because inhibition of export by Leptomycin B leads to a nuclear accumulation, however, at a much slower rate (almost by one order of magnitude). Thus, the rapid accumulation under cold stress is more likely to be caused by a stimulaton of import, rather than a block of export.

The association of OsDLK with cortical microtubules (Fig. 4a–c) is not unexpected for a plant kinesin. Some kinesins move cargoes linked with cellulose synthesis^41^, and also others that are not known to interact with cellulose synthases, nevertheless decorate cMTs. These include the class-XIV kinesins KCH and ATK5^7,11^. OsDLK moves along cMTs at high speed (Supplementary Movie S2), which might be linked with a function in cell growth.

Unexpected for a kinesin, OsDLK not only decorates cMTs, but simultaneously appears in the nucleus (Fig. 4d–j). This dual localisation was validated in three additional experimental systems using transient transformation: the leaf sheath of rice itself, protoplasts of *Arabidopsis*, and leaves of *Nicotiana benthamiana* (Fig. 5) In all four cases, the fluorescent signal was seen in the nucleus, although interphase microtubules are strictly excluded from the karyoplasm by the interphasic nuclear envelope^31,42^. However, this canonical strict exclusion of cytoskeletal proteins from the nucleus is progressively challenged by observations that kinesins can be found in the nucleus. For instance, certain animal KIF4s contain a NLS and are in fact found in the nucleus^43^ while AtFRA1 localizes only in cytoplasm^17^. Interestingly, a rice homologue of the exclusively cytoplasmic kinesin OsBC12 shown to localize in both cytoplasm and nucleus was found to be linked with a function as transcriptional regulator for a specific step in gibberellic acid (GA) biosynthesis^44^. When we probed this intranuclear population of OsDLK in more detail, we observed that cold-induced disassembly of cMTs was followed by a progressive accumulation of OsDLK that was organised in a reticular structure closely associated with chromatin and also tubulin (Fig. 8). This nuclear transport was reversible since cortical OsDLK recovered after cells were returned to room temperature. This behaviour parallels the cold-induced accumulation of tubulin in the nucleus observed in those cells^31^. However, even under normal temperature, there seems to be considerable recycling between the intranuclear and the cortical population of OsDLK, since treatment with the specific nuclear-export inhibitor Leptomycin B caused a progressive accumulation of the fluorescent signal in the nuclei of transgenic BY-2 cells (Fig. 9a), while the cortical signal was depleted (Fig. 9b–c).

### Conclusion and Perspectives

A rice member of the class-XIV kinesin family, OsDLK, has been found to cycle during interphase between the cortical MTs and the nucleus, and accumulates in the nucleus in response to cold. The same protein also conveys some functions during mitosis. These functions seem to overlap with those of the previously published homologues in *Arabidopsis* (ATK1 and ATK5). To get more insight into this mitotic function, a double visualisation with microtubules *in vivo* would be desirable, but this will require stable double transformation and selection of clones with balanced expression. The fact that nuclear transport of OsDLK occurs also under room temperature, but is promoted by cold stress, indicates that this kinesin plays a specific function in the nucleus. This function, at the current stage, is completely enigmatic. Preliminary data from rice insertion mutants indicate that OsDLK is essential for early development. This is supported by the observation that the steady-state levels of OsDLK transcripts are upregulated during coleoptile elongation. We have currently found that OsDLK can bind to DNA in a specific manner, however, whether it can exert transcriptional regulation similar to rice fra1 homologue is not clear. In other words: there is still a lot to be discovered, even for homologues of well-studied members of the kinesin superfamily.

## Materials and Methods

### Isolation and cloning of OsDLK

Rice (*Oryza sativa* L. *japonica* cultivar *Nipponbare*) seedlings were grown in darkness at 25 °C for 4 days and coleoptiles excised and shockfrozen in liquid nitrogen in aliquots of 100 mg, followed by grinding in a TissueLyser (Qiagen/Retsch Hilden, Germany). Total RNA was extracted with the innuPREP Plant RNA kit (Analytik, Jena, Germany), including on-column digest of genomic DNA with RNase-free DNAse I (Qiagen) according to the manufacturer instructions. After quality check by electrophoresis on 1% [w/v] agarose gels, 1 μg RNA was used for cDNA synthesis with the M-Mulv cDNA Synthesis Kit (NEB). Plasmids for plant transformation were constructed via GATEWAY^®^ cloning as described in Klotz and Nick^11^. The full-length coding sequence of OsDLK was amplified from the cDNA template with a pair of primers containing attB-sites. To get the full-length OsDLK the forward primer: 5′-GGGGACAAGTTTGTACAAAAAAGCAGGCTTCATGTCCACGCGCGCCACTCGCC-3′, and the reverse primer: 5′-GGGGACCACTTTGTACAAGAAAGCTGGGTCTCCTTGCGCCAAGCTACGCACT-3′ were used.

### Sequence analysis of OsDLK

The sequence motives and domains of OsDLK from *Oryza sativa* L*. japonica* were analysed by Prosite (http://prosite.expasy.org/cgi-bin/prosite) and SMART (http://smart.embl-heidelberg.de/smart). The neck region was predicted^27,45,46^. The software COILs (http://www.ch.embnet.org/software/COILS_form.html) was used^26^ to test the coiled coil regions. Candidate kinesins from different kinesin subfamilies of plants, animals and fungi, such as *Arabidopsis thaliana*, *Oryza sativa, Nicotiana tabacum, Gossypium hirsutum, Emericella nidulans, Drosophila melanogaster*, and *Saccharomyces cerevisiae* were aligned and then inferred into a phylogenetic tree by in MEGA5 (http://www.megasoftware.net/)^47^.

Accession numbers in UniProtKB/Swiss-Prot (http://www.uniprot.org) for the protein sequences used in the phylogenetic analysis can be accessed as follows: OsDLK(B8B6J5), DmNCD(P20480), EnKlpA(P28739), GhKCH1(Q5MNW6), NtTKRP125(O23826), NtKCH(F8UN41), ScKar3(P17119), OsACK1(Q9AWM8), OsKinesin13A(Q0DKM5), OsBC2(Q6YUL8), OsKCH(Q0IMS9), ATK5(F4JGP4), AtARK2(Q9LPC6), AtNACK1(Q8S905), AtARK3(Q9FZ06), AtKatA(Q07970), AtKinesin12B(F4J464), AtNACK2(Q8LNZ2), AtKCH(Q8W1Y3), AtKatB(P46864), AtPAKRP2(Q8VWI7), AtMKRP2(Q8W5R5), AtKCA1(Q9LX99), AtKatD(O81635), AtKatC(P46875), AtKAC2(Q9FKP4), AtKCBP(Q9FHN8).

### Plasmid construction

Using the GATEWAY^®^-Cloning technology (Invitrogen Corporation, Paisley, UK), the amplified PCR products of OsDLK were first recombined into the entry plasmid pDONR/Zeo (Invitrogen), and then cloned into the binary plasmid pK7FWG2 and pH7WGF2^48^ for stable and transient transformation, respectively. In these constructs, OsDLK was placed under control of the constitutive CaMV-35S promoter, and GFP was located C-terminally. Correct and complete insertion was verified by DNA sequencing (GATC Biotech, Cologne, Germany). Microtubules were visualised with the construct GFP-pCambiaTuB6^49^, in which GFP was fused to *Arabidosis* β-Tubulin 6. The OsDLK bacterial expression construct was amplified using the primers 5′-CACAGCAGCGGCCTGGTGCCGACTCGCCCCGGGATGCTCCACCAGAAG and 5′-CTTTCGGGCTTTGTTAGCAGCCGGATCTCATCCTTGCGCCAAGCTACGCACTTGGG and inserted into the pET28a bacterial expression vector (Novagen) via overlap extension cloning^50^.

### Transformations and cell culture

pK7FWG2-OsDLK was transformed into BY-2 (*Nicotiana tabacum* L. cv. *Bright Yellow 2*) cells via the *Agrobacterium* strain LBA4404 (Invitrogen) for establishment of stable overexpression cell line based on standard protocol^51^ with minor modifications described by Klotz and Nick^11^. Cell suspension cultures were then maintained in liquid Murashige and Skoog (MS) medium and subcultured weekly^52^. For transient transformation^53^, pH7RWG2-OsDLK and GFP-pCambiaTuB6 were co-transformed into LBA4404, subsequently grown on solid Paul’s medium for 3 days, and then examined microscopically without preceding selection.

Transient protein expression in mesophyll protoplast of *Arabidopsis thaliana* was performed as described in by Tong *et al*.^54^. Protoplasts were incubated for 24 hours at room temperature in the dark. Also transient protein expression in *Nicotiana benthamiana* leaves was carried out according Tong *et al*.^54^ via *Agrobacterium tumefaciens* C58C1 strains^55^ harbouring the pK7FWG2-OsDLK construct or a construct for the tobamovirus RNA silencing suppressor p19^56^. Rice (*Oryza sativa* L. *japonica* cultivar *Nihonmasari*) seedlings were grown in phytoagar at 25 °C for 10 days^57^. pK7FWG2-OsDLK DNA was transformed into the second leaf blades following the protocol by Holweg *et al*.^53^. The transformed rice blades were incubated in the dark at 25 °C for 24 h before examination by microscopy.

### *In vivo* microscopy and image analysis

Cellular details of individual cells or cells population were examined under an AxioObserver Z1 microscope (Zeiss, Jena, Germany) equipped with a cooled digital CCD camera (AxioCam MRm) and a spinning-disc device (YOKOGAWA CSU-X1 5000) or an ApoTome microscope slider for optical sectioning. GFP fluorescence and RFP/TRITC fluorescence signals were observed through the 488 nm and 561 nm emission line of an Ar-Kr laser (Zeiss), DNA labelling by Hoechst 33258 was recorded using the filter set 49 DAPI^11^. To detect fluorescence of DLK-GFP in living *N. benthamiana* leaf cells and *A. thaliana* protoplasts, confocal laser scanning microscopy was applied by using the Leica TCS SP8 Confocal Platform (Leica Microsystems, Wetzlar, Germany). The detection windows ranged from 496 to 511 nm (GFP), 569 to 591 nm (RFP), as well as 690 to 708 nm for the detection of chlorophyll autofluorescence. Acquired images were operated via the Zen 2012 (Blue edition) software platform, AxioVision (Rel. 4.8.2) software and ImageJ (NIH, Bethesda, USA).

### Determination of packed cell volume

To quantify culture growth, packed cell volume (PCV) was measured at days 4 (after the proliferation phase) and 6 (at the end of expansion phase) after subcultivation^58^. The cell suspension was poured into a 15-mL Falcon tube and kept vertically at 4 °C for 48 hours, till most cells had settled to the bottom. The PCV could then be read directly from the scale of the 15-mL Falcon tube.

### Determination of mitotic index

Tobacco BY-2 cell cycle progress was monitored by mitotic indices (MI), defined as the relative frequency of dividing cells. BY-2 cells were fixed by Carnoy fixative and Hoechst 33258 (Sigma, Taufkirchen, Germany) at a final concentration of l μg·mL^−1^, and samples were immediately investigated with microscope.

### Cell cycle synchronization

Cells were synchronized according to a protocol modified from Samuels *et al*.^59^ by using hydroxyurea instead of aphidicolin^60^. 7-d-old BY-2 cells were subcultured in MS medium complemented with 4 mM hydroxyurea (HU, Sigma) for 24 h. After 24 hours, HU was washed out by a Nalgene filter holder (Thermo Scientific, Langenselbold, Germany) in combination with a Nylon mesh with pores of diameter of 70 μm (Mehlsieb, Franz Eckert, Waldkirch, Germany). Then the cells were resuspended in fresh MS medium for cultivation, returned into flask and shaken for a further 3 h. 6 μM Propyzamide (Sigma) was added into the culture and the suspension was shaken for another 6 h. Subsequently, propyzamide was removed. After washing, cells were resuspended again in fresh MS medium and cultured on a shaker, while MI was monitored every 30 min over 4 hours.

### Immunostaining of microtubules

Microtubules were visualized by indirect immunofluorescence as described in Nick *et al*.^61^ with mouse monoclonal antibodies against α-tubulin, DM1A (Sigma) and a polyclonal secondary TRITC-conjugated anti-mouse IgG antibody (Sigma). In some cases, the DNA was also stained with Hoechst 33258.

### Cold treatment and cell mortality

Suspensions of 3-day-old BY-2 cells overexpressing OsDLK-GFP in Erlenmeyer flasks were placed in a bath of ice water to maintain a temperature of 0 °C and shaken on an orbital shaker at 100 rpm in darkness for 24 h. Samples of cells were collected at specified time points during the cold treatment for cytological observation and immunostaining. Cell mortality was quantified using Evans Blue assay^62^. After remove the dye by washing 3 times in water, cells were viewed under AxioImager Z.1 microscope (Zeiss). Data represent means and standard deviations from three biological replicates with 500 individual cells scored for each value.

### Leptomycin B treatment

To measure the accumulation of OsDLK in nuclei, OsDLK-GFP BY-2 cells in their exponential phase of growth (3 days after inoculation) were treated with 200 nM Leptomycin B (Sigma), an inhibitor of nuclear export. The cells were incubated for a further 48 h under standard conditions, and z-stacks of GFP signal were recorded (AxioImager Z.1). For the quantification, geometrical projections (maximum intensity algorithm) were quantified using Image J. Intensity profiles along a very broad probing line (in the thickness of roughly the nucleus) were collected across the entire cross section of the cell and then a second time along the same plane, but just covering the nucleus. The two integral over these two profiles were used to calculate the percentage of signal located inside the nucleus. Control treatments were performed by treating the cells with the corresponding volume of solvent [70% methanol].

### Protein expression and purification

C-terminally hexa-histidine-tagged OsDLK(aa 1–764) was expressed in *Escherichia coli* BL21(DE3)-pRARE (Millipore) grown in LB medium and induced with 0.2 mM IPTG for 3 h at 37 °C. Harvested cells were resuspended in buffer A (pH 7.4, 274 mM NaCl, 5.4 mM KCl, 16.2 mM Na_2_HPO_4_, 3.52 mM KH_2_PO_4_, 2 mM MgCl_2_, 1 mM ATP, 1 mM dithiothreitol, and EDTA-free protease inhibitors (Roche)) and lysed using a high pressure homogenizer. The crude lysate was centrifuged at 17,400 g at 4 °C and loaded onto a 5 mL HiTrap NiNTA column (GE Healthcare). The column was washed with 50 mL buffer A containing 30 mM imidazole. Proteins were eluted in buffer A containing 500 mM imidazole, pH 8.0. Proteins were snap-frozen in liquid nitrogen and stored at −80 °C.

### Motility assays *in vitro*

Microtubules were polymerized as described before using DyLight594-labeled or a mixture of Cy5-labeled and digoxygenin-labeled (1:5) tubulin. For the microtubule sliding motility assay, microtubules co-labeled with digoxygenin and Cy5 were immobilized to the glass surface via digoxygenin antibodies (Roche). After blocking with 1% Pluronic F127, OsDLK motors were added to the microtubules in absence of ATP. Subsequently, microtubules labeled with DyLight594 in imaging solution were allowed to bind to the motors and transport was monitored in presence of 2 mM ATP. Fluorescently labeled microtubules were visualized using epi-illumination on an inverted fluorescence microscope (Ti-E, Nikon) equipped with an EMCCD camera (iXon Ultra, Andor). Positions of microtubules were obtained using FIESTA tracking software as described before^63^. The mean velocity was determined by fitting the velocity histograms to Gaussian functions using MatLab (Mathworks).

### Data availability

The datasets generated during and/or analysed in the current study are available from the corresponding author on reasonable request.

## Electronic supplementary material

Supplementary Figure S1-S7

Supplementary Movie S1

Supplementary Movie S2
