# Supplementary material for: A rice class-XIV kinesin enters the nucleus in response to cold
Source: Sci Rep. 2018 Feb 26;8:3588. doi: 10.1038/s41598-018-21816-w (PMC5827730; doi:10.1038/s41598-018-21816-w)
Supplement: Supplementary file 1 — Supplementary Figure S1-S7 [file 41598_2018_21816_MOESM1_ESM.doc]

**A rice class-XIV kinesin enters the nucleus in response to cold**

Xiaolu Xu^a,*^, Wilhelm J.Walter^b^, Qiong Liu^a^, Isabel Machens^b^, Peter Nick^a^

**^a^** *Molecular Cell Biology, Botanical Institute, Karlsruhe Institute of Technology (KIT), Fritz-Haber-Weg 4, 76131 Karlsruhe, Germany.*

**^b^** *Molecular Plant Physiology, Biocentre Klein Flottbek, University of Hamburg, 22609 Hamburg, Germany.*

**Corresponding author: Xiaolu Xu, Molecular Cell Biology, Botanical Institute, Karlsruhe Institute of Technology (KIT), Fritz-Haber-Weg 4, 76131 Karlsruhe, Germany.*

*fax +49 721 608 44193, e-mail xiaolu.xu@student.kit.edu*

*Email address: xu.xiaolu@hotmail.com; wim.walter@uni-hamburg.de; liuqionglandmail@gmail.com; isabel.machens@uni-hamburg.de; peter.nick@kit.edu*

**
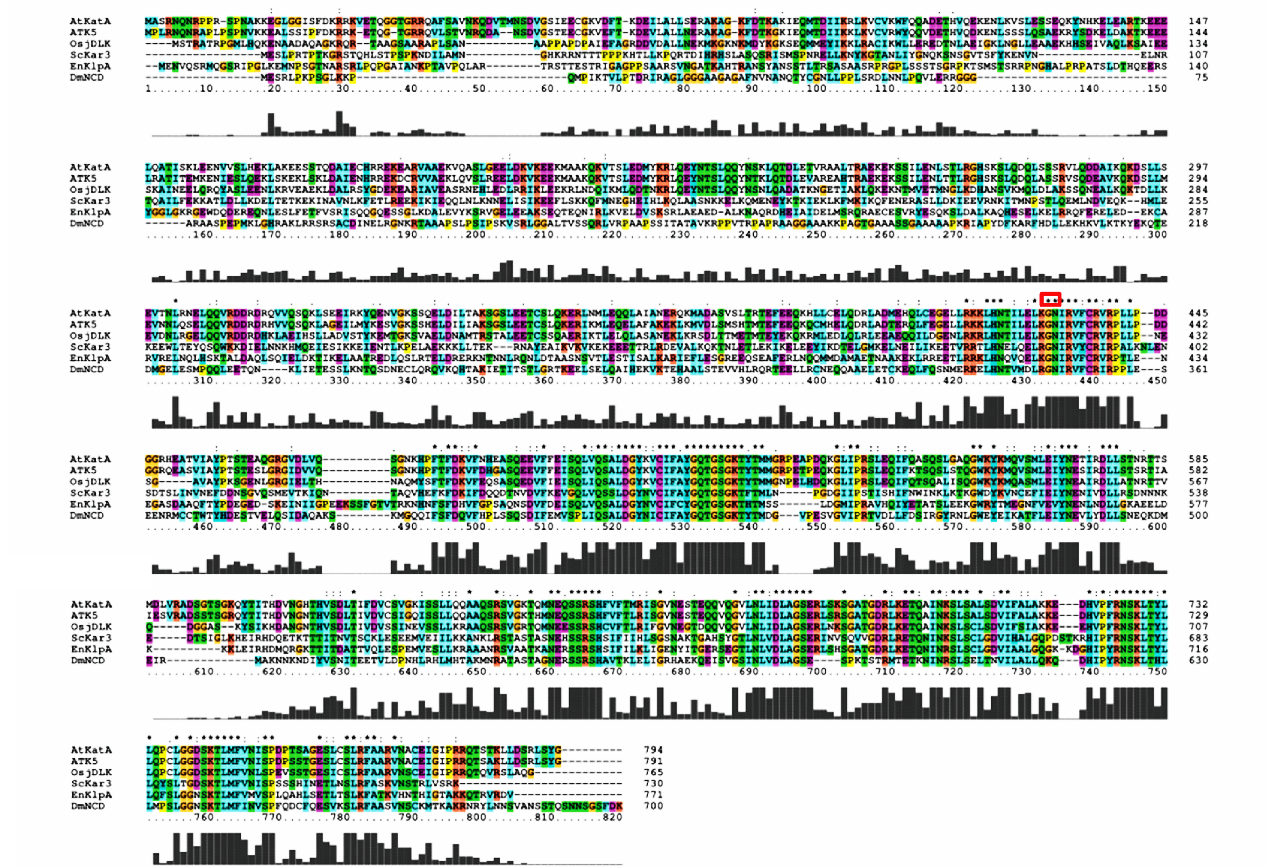
**

**Fig. S1:** Alignment of OsDLK and other members of kinesin AtKatA, ATK5, DmNCD, ScKar3, En KlpA. An amino-acid sequence alignment of the putative neck linker region of OsDLK and related kinesins shows that the 14-aa stretch (marked by rectangle) associated with minus-end directionality in the kinesin-14 family is partially conserved. Small rectangle indicates the two amino acids known to by mostly connected with kinesin minus-end directed movement.


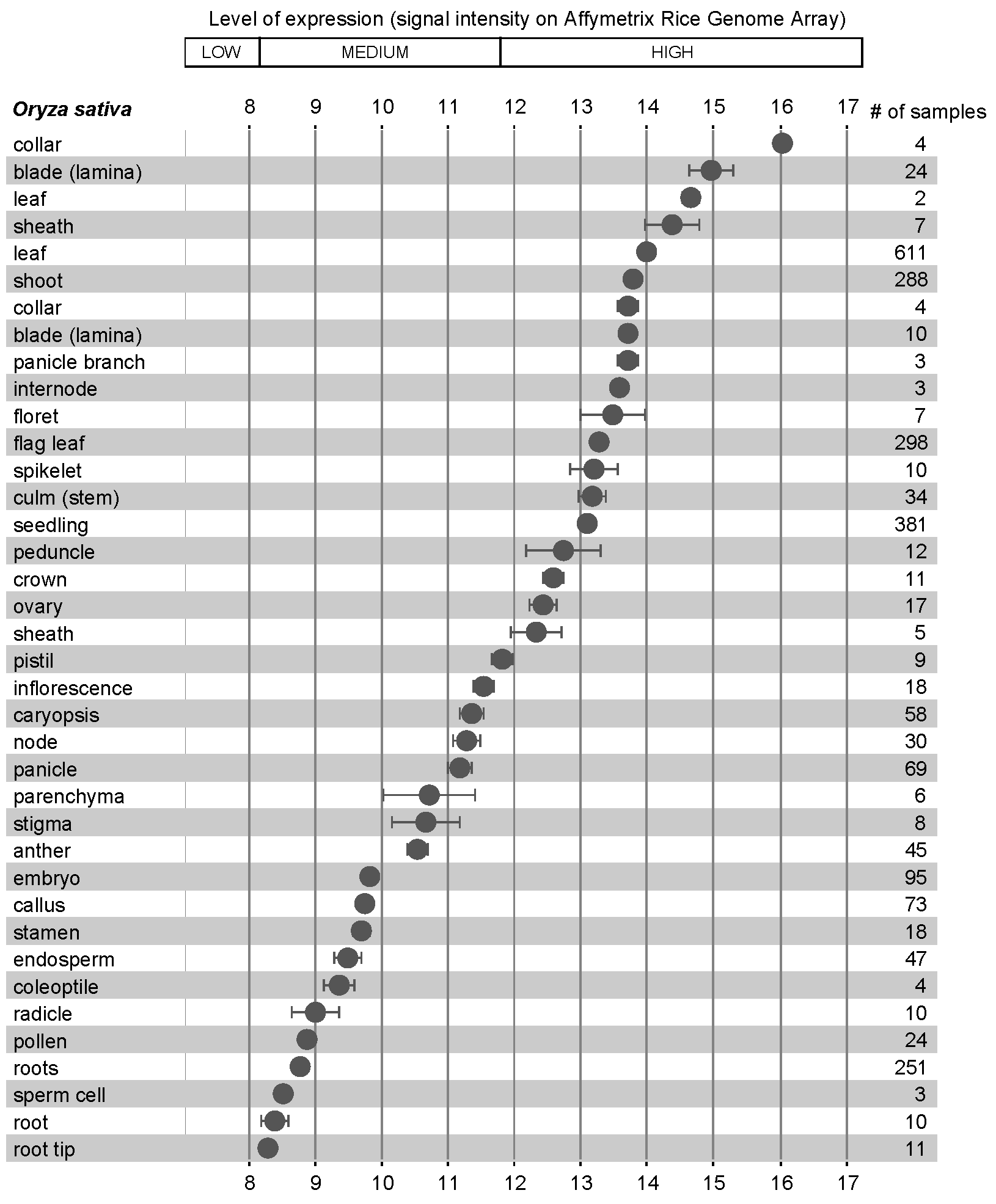


**Fig. S2:** Scatter plot of tissue-dependent OsDLK transcription levels in different tissues generated with the *Genevestigator* software (NEBION, Switzerland) from an Affymetrix Rice Genome Array database^30^. The error bars represent the SEM for the indicated number of independent samples.


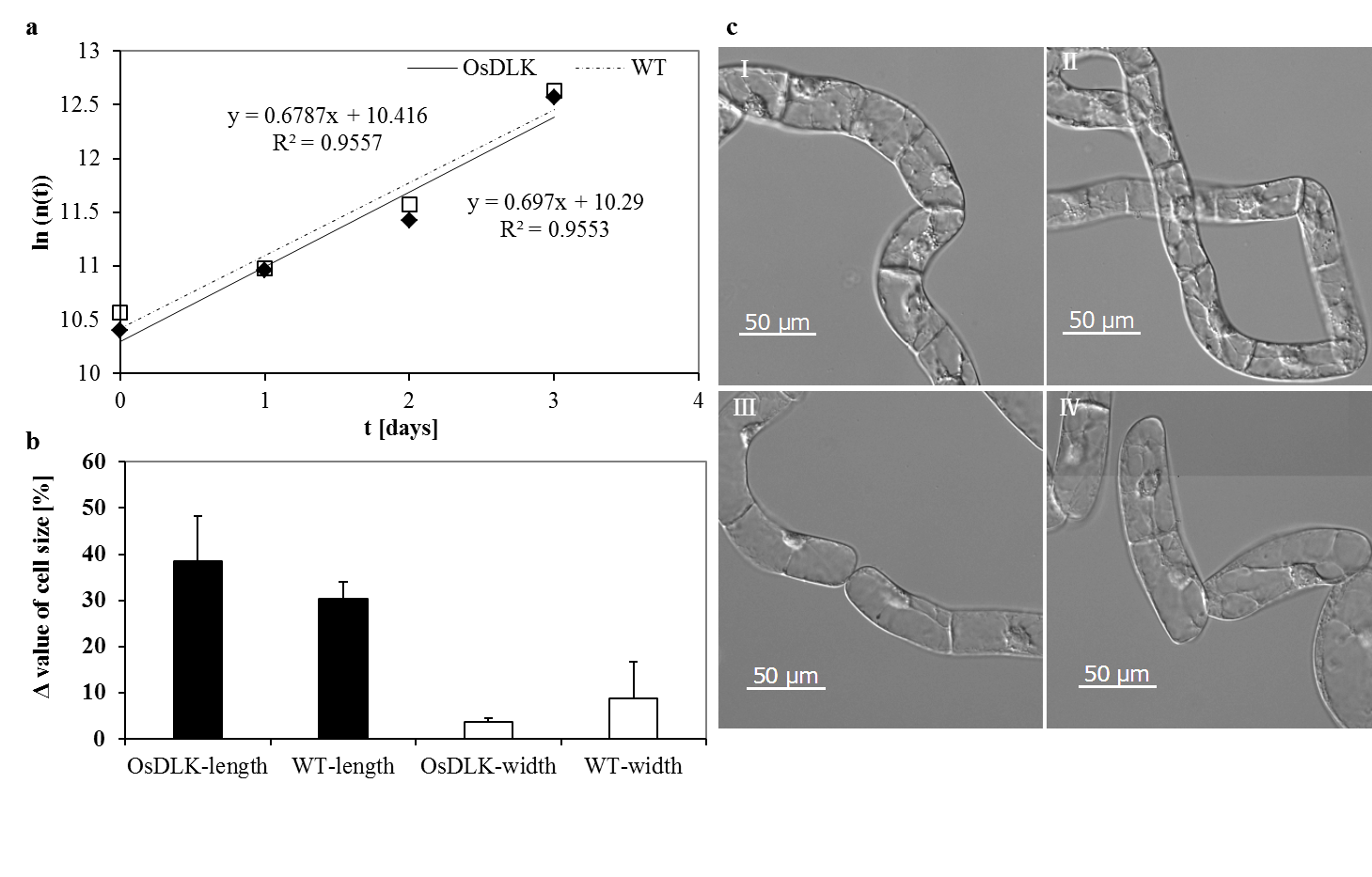


**Fig. S3:** (a) Estimated cell cycle lengths for OsDLK-GFP and non-transformed BY-2 WT. (b) Promoted cell elongation during the expansion phase of late cultivation cycle in the OsDLK-GFP overexpressor versus the non-transformed WT. Error bars represent the standard errors of triplicate measurements. (c) Representative cell files of the OsDLK-GFP overexpressor (I, III) compared to the non-transformed wildtype (II, IV) at day 3 (I, II) and at day 7 (III, IV); (I) OsDLK-GFP at day 3; (II) WT at day 3; (III) OsDLK-GFP at day 7; (IV) WT at day 7.


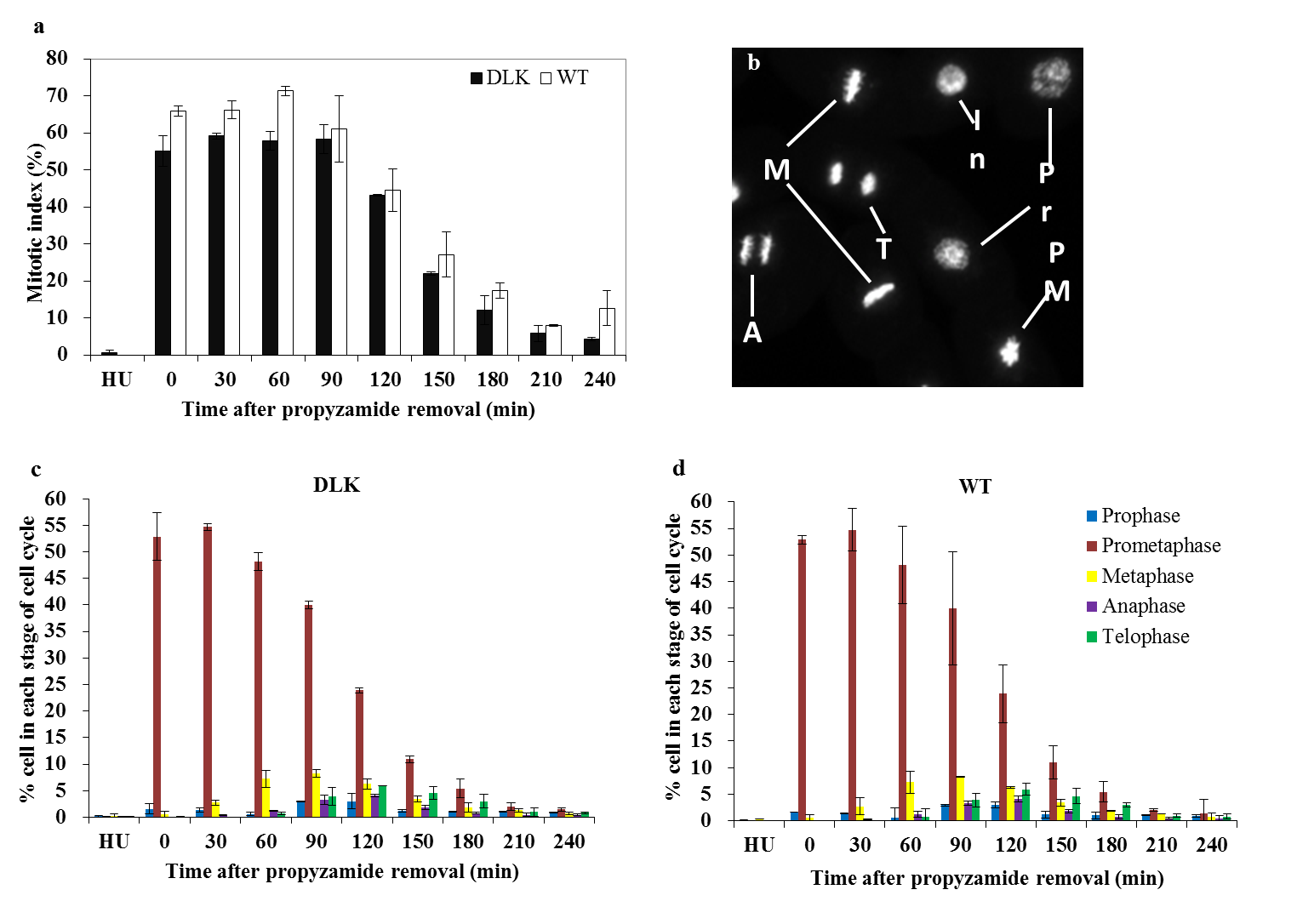


**Fig. S4:** Mitotic synchronisation in the OsDLK-GFP overexpressor (white bars) as compared to the non-transformed wildtype (black bars). (a) Time course of mitotic index (MI) after synchronisation with hydroxyurea (HU) for 24 h and with propyzamide for 3 h. Time points represent the interval after release from propyzamide treatment. (b) Representative image of cells stained for DNA after release from propyzamide. In, Interphase cells; Pr, Prophase cell; PM, Prometaphase cells; M, Metaphase cells; A, anaphase cells; T, Telophase cells. (c) Time course for the frequency of individual mitotic stages following release from propyzamide in OsDLK-GFP and non-transformed WT BY-2. Error bars represent the standard error of from biological triplicates comprising a population of 3000 cells per data point.


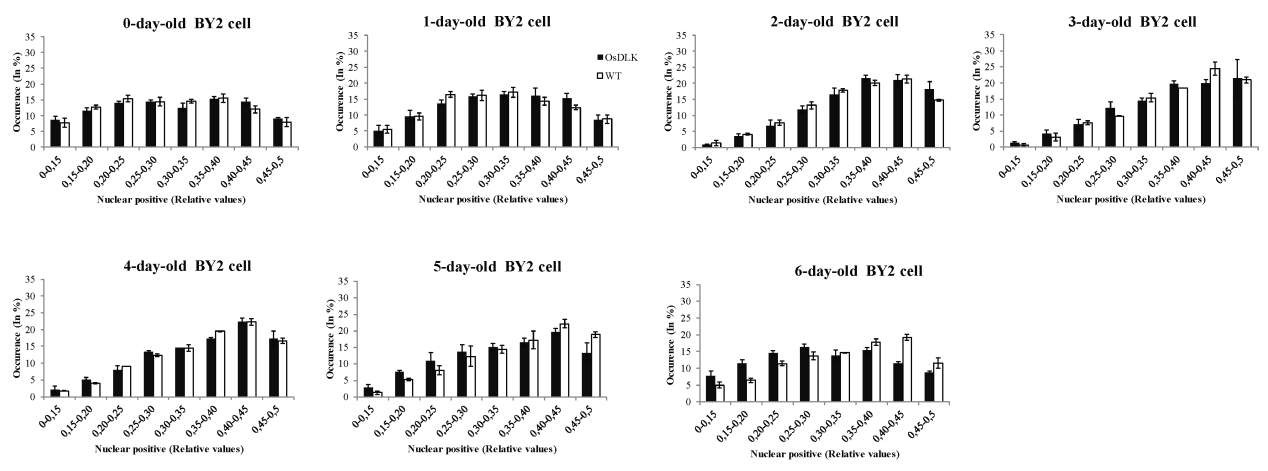


**Fig. S5:** Time course of nuclear positioning. Frequency distributions of nuclear position were recorded in OsDLK-GFP BY-2 (black bars) and the non-transformed WT (white bars) at daily intervals over the entire cultivation cycle. A value of 0.5 represents a position in the cell center, a value of 0 represents a position at the lateral walls. Error bars represent the standard error from biological triplicates comprising a population of 1500 cells per data point.


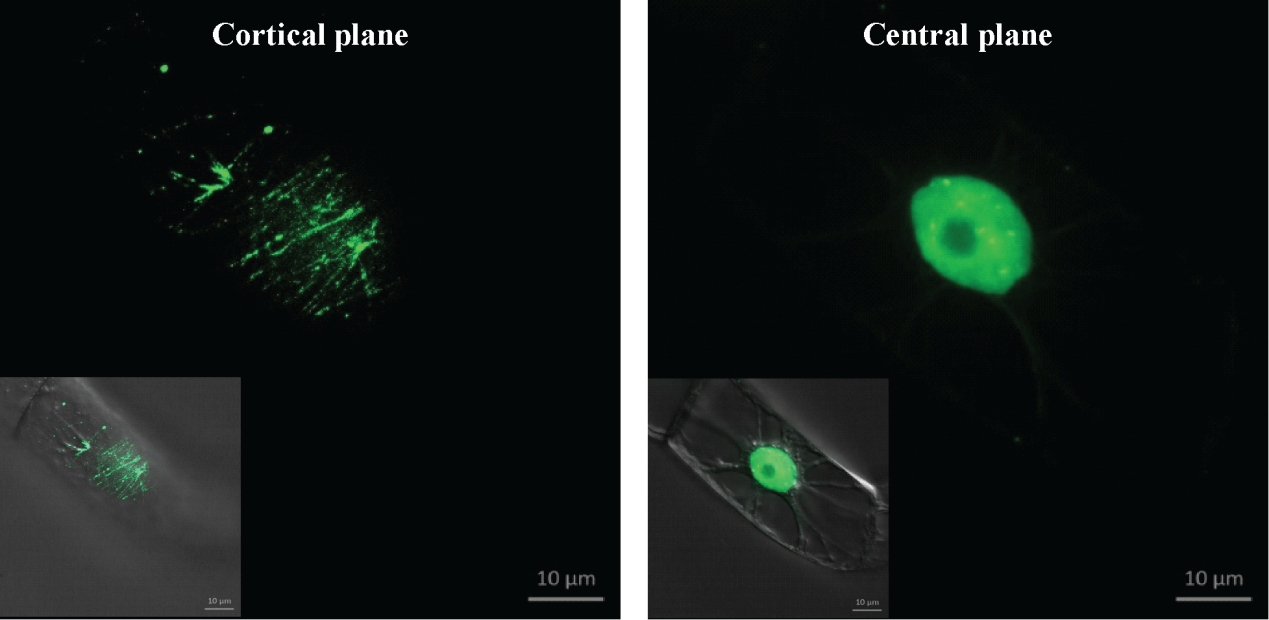


**Fig. S6:** OsDLK-GFP is exported from nucleus during recovery from cold treatment. Confocal sections in the cortical and the nuclear plane of OsDLK-GFP expressing cells that had been subjected to cold stress for 7 hours, and were then allowed to recover at 25°C for 6 hours. Scale bars: 10 μm.


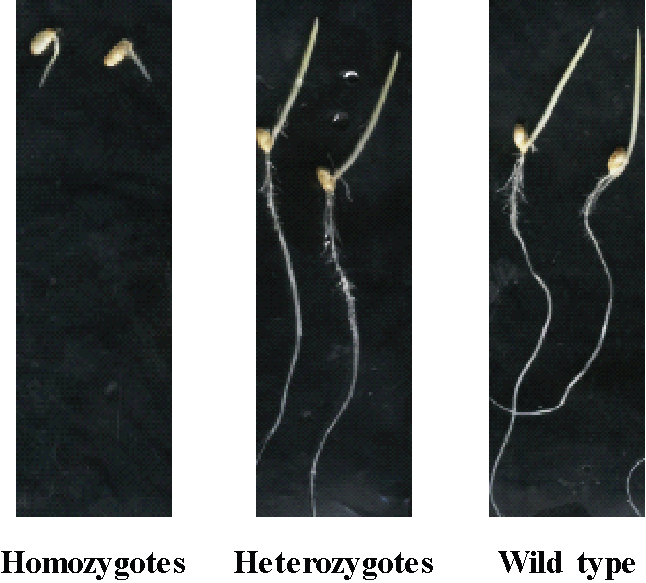


**Fig. S7:** A T-DNA insertion rice mutant of OsDLK is impaired in seed germination. Representative images of etiolated seedlings grown for 6 d at 25°C. While wild type and heterozygous plants show a fully elongated coleoptile and a well developed seminal root, the homozygote has only produced a short and crippled coleoptile and fails to generate any root at all. These seedlings all die during the subsequent days.
